# BODIPY derivatives with near infra-red absorption as small molecule donors for bulk heterojunction solar cells†

**DOI:** 10.1039/c9ra01750j

**Published:** 2019-05-16

**Authors:** John Marques dos Santos, Lethy Krishnan Jagadamma, Najwa Mousa Latif, Arvydas Ruseckas, Ifor D. W. Samuel, Graeme Cooke

**Affiliations:** School of Chemistry, University of Glasgow Joseph Black Building Glasgow G12 8QQ UK Graeme.Cooke@glasgow.ac.uk; Organic Semiconductor Centre, SUPA, School of Physics and Astronomy, University of St. Andrews St. Andrews, Fife KY16 9SS UK idws@st-andrews.ac.uk

## Abstract

The study of small donor molecules as the active component of organic solar cells continues to attract considerable attention due to the range of advantages these molecules have over their polymeric counterparts. Here we report the synthesis and solar cell fabrication of three BODIPY small molecule donors. Two of the dyes feature triphenylamine and phenothiazine as donor units attached to the *meso* and α-positions of the BODIPY core (TPA-PTZ-DBP and PTZ-TPA-BDP). Additionally, we have synthesised a push–pull derivative featuring phenothiazine moieties in the α-positions and a nitrobenzene in the *meso*-position (N-TPA-BDP) in order to investigate what effect this type of functionalisation has on the photovoltaic properties compared to the other dyes. The optoelectronic properties were investigated and the dyes showed broad absorption in the near-infrared with high extinction coefficients. Electrochemical measurements indicated good reversibility for the dyes redox processes. In contrast with the all-donor functionalised systems, N-TPA-BDP demonstrated extensive HOMO–LUMO overlap by DFT. The dyes were investigated as donor molecules in bulk heterojunction solar cells along with PC_71_BM, and under optimal donor to acceptor ratio PTZ-TPA-BDP showed the highest PCE of 1.62%. N-PTZ-BDP:PC_71_BM was the only blend to further improve upon thermal annealing reaching the highest conversion efficiency among the dyes of 1.71%. A morphology comprised of finely mixed donor and acceptor components is observed for BHJ blends of each of the three donors at their optimum fullerene content. Upon thermal annealing, these morphological features remain mostly the same for PTZ-TPA-BDP:PC_71_BM and TPA-PTZ-DBP:PC_71_BM blends whereas for N-PTZ-BDP:PC_71_BM the domains show a larger size. These dyes show that phenothiazine functionalisation of BODIPY is useful for solar cells because it gives strong and broad absorption extending to the near infra-red and materials with reversible redox properties – both of which are desirable for organic solar cells.

## Abstract 2

We report the synthesis of donor/acceptor functionalised BODIPY derivatives and their incorporation as donor molecules in bulk heterojunction solar cells.

## Graphical abstract

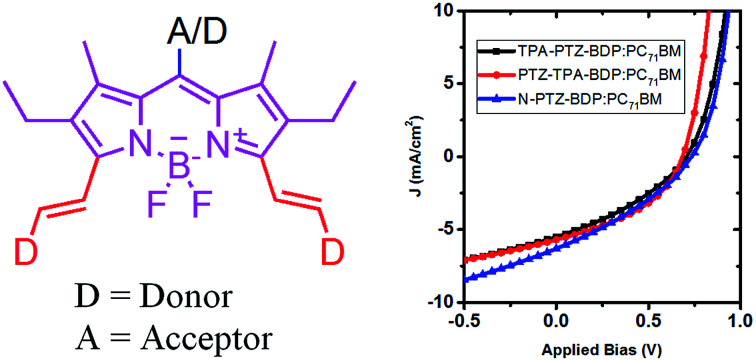

## Introduction

Organic electronic materials have undergone considerable development and possess several attractive features for applications such as transistors,^1^ light emitting diodes^2^ and solar cells.^3,4^ Organic photovoltaic (OPV) materials have attracted considerable attention due to their low cost, flexibility, high optical absorption coefficient, and simple fabrication.^5,6^ To obtain high performance and good stability, OPV materials need to have broad absorption, enable efficient charge generation and extraction whilst also having reversible redox processes.^7^ Enormous effort has been directed towards device engineering and optimization of the chemical structure of active materials which has resulted in ever increasing efficiency of both polymer and small molecule solar cells.^8–10^ Bulk heterojunction (BHJ) solar cells are fabricated from a blend of an electron donor (D) and an electron acceptor (A),^11^ and consist typically of a low bandgap polymer or small molecule. One of the many advantages of organic solar cells is that for many materials the devices can be fabricated using solution-processing techniques, which favour large-scale production, and have led to organic solar cells achieving power conversion efficiencies (PCE) > 10%.^4,12,13^ Small molecules continue to be very attractive candidates for organic solar cells as they can be typically synthesized in large scale with reproducible and well established protocols and are becoming competitive with their macromolecular brethren in terms of power conversion efficiencies.^5^

The design of active materials in OPV research is an ongoing challenge in order to produce small molecules that possess strong optical absorption characteristics and appropriate orbital offset to favour light harvesting, exciton diffusion and promote high charge carrier mobility.^12,14^ Boron-dipyrromethene (BODIPY) dyes have emerged in recent years as interesting dyes for OPVs because they possess many of these properties. In addition, BODIPYs usually possess good chemical, photochemical and thermal stability, and often feature deep HOMO energy levels which lead to a high open circuit voltage (*V*_oc_).^15,16^ Several strategies have been taken to modify the BODIPY core and tune the properties in the desired direction.^14–24^ Diverse molecular structures have been investigated by attaching donor moieties to different positions of the BODIPY core giving molecules with near IR absorption, high molecular extinction coefficient (*ε*),^15,19,22,25–27^ near-planar molecular structure,^14,17,28^ efficient charge transport and good quality film formation,^16,17,19,28^ together with favourable power conversion efficiencies (PCEs).^29^ However, despite the remarkable photo-physical properties and the promising results reported so far,^29–31^ understanding the role functional units attached to the BODIPY core play in determining their optoelectronic and photovoltaic properties remains an important goal in order to understand the best approach to combine donor and acceptor units within these dyes to produce different BODIPY based BHJ solar cells.

**Fig. 1:**
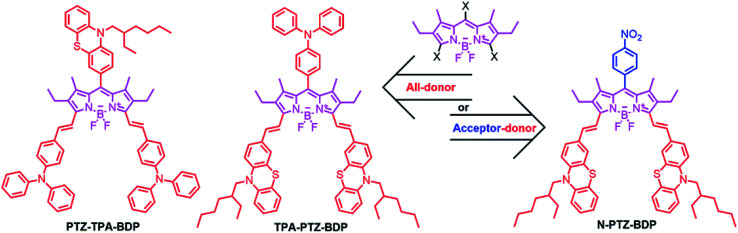
Chemical structures of the BODIPY derivatives used in this study.

The incorporation of donor moieties on the 3,5-positions (α) of the BODIPY core appears to be the preferred approach to avoid highly twisted structures.^14,19–22^ In fact, one of the best performing BODIPY dyes possessing a highly planar molecular structure is functionalized with vinyl-thiophene units attached to these positions.^14^ On the other hand, Mao *et al.* recently indicated that phenothiazine (PTZ) donor moieties communicate adequately with the BODIPY core through the 2,6-positions which leads to some of the best performing BODIPY-based dye sensitized solar cells,^32,33^ presumably due to the electron donating ability and near-planar butterfly conformation of the PTZ unit.^34^ Interestingly, Liao *et al.* recently used this moiety to fabricate a 2,6-linked dimer for BHJ solar cells that achieved a 3.33% PCE.^17^ Therefore, incorporation of these moieties in the α-positions is attractive as α-substituted BODIPYs possess some of the most attractive properties from this family of dyes for OPVs (including near-IR absorption and high absorptivity).^16,35^ In the present work, we report the synthesis and characterization of three new BODIPY dyes functionalized in the *meso* and α-positions for application as donor material in BHJ solar cells. We have investigated the effect of adding three donor units [phenothiazine (PTZ) and triphenylamine (TPA)] to the *meso* and α-positions (abbreviated from hereafter as PTZ-TPA-BDP and TPA-PTZ-BDP) and in the case of N-PTZ-BDP the addition of an electron accepting nitrobenzene unit to the *meso*-position of the BODIPY core has on the optical, electronic and BHJ solar cell device characteristics (Fig. 1).

## Experimental section

### General synthesis

All the reagents were purchased from Sigma Aldrich®, Fluorochem®, TCI®, Alfa Aesar®, Acros® or Fisher Scientific® and used as received. Column chromatography was carried out using silica gel (Sigma-Aldrich) 40–63 μm particle size (60 Å pore size). Thin-layer chromatography (TLC) was performed using Merck silica gel 60 covered aluminium plates F254. Dry solvents were obtained from Innovative Technology inc. Pure Solv 400-5-MD solvent purification system (activated alumina columns) or Sigma Aldrich®. Melting points (Mp) were recorded on a SMP-10 Stuart Scientific melting point machine and are uncorrected.

NMR spectra were obtained using either a Bruker Avance III 400 or a Bruker Avance III 500 spectrometers. Mass spectra were obtained from the mass spectrometry service at the University of Glasgow. All the spectroscopic and electrochemical data were processed using the Origin Pro 8.5 software suite.

### Synthesis of 3^36^

10*H*-Phenothiazine (1) (5.00 g, 25.0 mmol) and potassium *tert*-butoxide (3.50 g, 31.2 mmol) were dissolved in dry THF (30 mL) and the solution was stirred under an argon atmosphere for 1 h. 2-Ethylhexyl bromide (2) (4.5 mL, 4.9 g, 25 mmol) was added dropwise and the reaction mixture was stirred under reflux for 24 h. The mixture was cooled to room temperature, washed with brine (100 mL), extracted with DCM (2 × 100 mL), dried over MgSO_4_ and filtered. After evaporation of the solvent under reduced pressure, flash column chromatography was performed over silica gel using petroleum ether as eluent to yield the desired product 3 as a colourless oil (6.53 g, 83%). ^1^H NMR (400 MHz, CDCl_3_) *δ* 7.21–7.12 (m, 4H), 6.91 (m, 4H), 3.75 (d, *J* = 7.1 Hz, 2H), 2.04–1.88 (m, 1H), 1.59–1.35 (m, 4H), 1.33–1.20 (m, 4H), 0.92–0.85 (m, 6H); ^13^C NMR (101 MHz, CD_3_COCD_3_) *δ* 146.9, 128.3, 128.2, 126.5, 123.4, 117.2, 51.6, 36.9, 31.4, 29.3, 24.7, 23.8, 14.4, 11.0; *m*/*z* (ESI^+^) 311.1702 [M^+^] (C_20_H_25_NS requires 311.1708).

### Synthesis of 4^36^

POCl_3_ (20.0 mL, 32.8 g, 0.21 mol) was added slowly into DMF (16.0 mL, 15.1 g, 0.20 mol) at 0 °C under an argon atmosphere. The reaction mixture was stirred for 1 h. The mixture was slowly added to a solution of compound 3 (6.50 g, 20.9 mmol) in 1,2-dichloroethane (100 mL) at 0 °C. After complete addition, the mixture was stirred at 90 °C for 24 h under an argon atmosphere. The reaction mixture was cooled to room temperature, neutralized with a saturated solution of K_2_CO_3_ (100 mL) and extracted with DCM (3 × 100 mL). The organic layer was washed with water (300 mL), dried over MgSO_4_ and filtered. After evaporation of the solvent under reduced pressure, flash column chromatography was performed over silica gel (petroleum ether : DCM; 8 : 2) to yield the desired product 4 as a yellow oil (6.48 g, 91%). ^1^H NMR (400 MHz, CDCl_3_) *δ* 9.80 (s, 1H), 7.69–7.59 (m, 2H), 7.17 (m, 2H), 7.03–6.87 (m, 3H), 3.85–3.76 (m, 2H), 2.02–1.84 (m, 1H), 1.51–1.33 (m, 4H), 1.32–1.17 (m, 4H), 0.86 (m, 6H); ^13^C NMR (101 MHz, CDCl_3_) *δ* 190.2, 151.6, 144.1, 131.2, 129.9, 128.8, 127.9, 127.6, 126.3, 125.0, 123.7, 116.7, 115.6, 51.5, 36.1, 30.7, 28.6, 24.0, 23.1, 14.1, 10.5; *m*/*z* (ESI^+^) 362.1549 [M + Na^+^] (C_21_H_25_NOSNa requires 362.1555).

### Synthesis of 8

2,4-Dimethy-3-ethyl pyrrole (5) (1.1 mL, 8.1 mmol) and compound 4 (1.38 g, 4.06 mmol) were dissolved in argon-degassed DCM (300 mL). Three drops of trifluoroacetic acid were added and the mixture was stirred for 24 h at room temperature. During this time the solution changed from pale yellow to a deep red colour. *p*-Chloranil (1.00 g, 4.06 mmol) was then added to the mixture at 0 °C and the reaction was allowed to stir for 3 h. *N*,*N*-diisopropylethylamine (8.0 mL, 45 mmol) was added to the mixture dropwise at room temperature over a period of 5 min, and the solution was stirred for 30 min. Subsequently, BF_3_·OEt_2_ (8.0, mL, 64.8 mmol) was added dropwise at 0 °C over a period of 10 min, and the mixture was stirred overnight at room temperature. The reaction mixture was washed with water (50 mL), extracted with DCM (2 × 50 mL), dried over MgSO_4_ and filtered. After evaporation of the solvent under reduced pressure, flash column chromatography was performed over silica gel (DCM : petroleum ether; 4 : 1) to yield the desired product 8 as a red solid (1.58 g, 63%). Mp: 147–149 °C; ^1^H NMR (400 MHz, CDCl_3_) *δ* 7.23–7.10 (m, 2H), 7.04–6.99 (m, 2H), 6.98–6.89 (m, 3H), 3.78 (d, *J* = 7.2 Hz, 2H), 2.52 (s, 6H), 2.30 (q, *J* = 7.5 Hz, 4H), 2.03–1.90 (m, 1H), 1.49–1.37 (m, 4H), 1.40 (s, 6H), 1.27 (q, *J* = 7.6, 4.6 Hz, 4H), 0.98 (t, *J* = 7.6 Hz, 6H), 0.88–0.84 (m, 6H); ^13^C NMR (101 MHz, CDCl_3_) *δ* 153.7, 145.9, 145.7, 139.5, 138.4, 132.8, 131.1, 129.4, 127.6, 127.4, 127.3, 127.1, 126.9, 125.2, 122.8, 116.5, 116.1, 51.4, 35.9, 30.7, 28.7, 24.0, 23.1, 17.2, 14.7, 14.1, 12.6, 12.2, 12.1, 10.6; *m*/*z* (FAB^+^) 613.3469 [M^+^] (C_37_H_46_^11^BF_2_N_3_S requires 613.3479).

### Synthesis of TPA-PTZ-BDP

Compounds 7 (0.80 g, 1.46 mmol) and 4 (2.48 g, 7.31 mmol) were dissolved in dry toluene (30 mL) containing activated molecular sieves (80 mg, 4 Å). Acetic acid (6.4 mL) and piperidine (6.4 mL) were added into the solution and the mixture was stirred under an argon atmosphere under reflux until consumption of the starting material was confirmed by TLC. The mixture was cooled to room temperature and the solvent was evaporated under reduced pressure. Subsequently, the reaction mixture was washed with brine (50 mL), extracted with DCM (3 × 50 mL), dried over MgSO_4_ and filtered. After evaporation of the solvent under reduced pressure, flash column chromatography was performed over silica gel (CHCl_3_ : petroleum ether; 1 : 1) to yield the desired product TPA-PTZ-BDP as a dark green solid (0.143 g, 8%). Mp: 245–247 °C; ^1^H NMR (400 MHz, CDCl_3_) *δ* 7.65 (d, *J* = 16.7 Hz, 2H), 7.48 (dd, *J* = 8.5, 1.9 Hz, 2H), 7.37 (d, *J* = 1.9 Hz, 2H), 7.34–7.28 (m, 4H), 7.20–7.13 (m, 14H), 7.10–7.04 (m, 2H), 6.97–6.87 (m, 6H), 3.78 (d, *J* = 7.1 Hz, 4H), 2.62 (q, *J* = 7.4 Hz, 4H), 2.04–1.91 (m, 2H), 1.54 (s, 6H), 1.52–1.36 (m, 8H), 1.33–1.25 (m, 8H), 1.17 (t, *J* = 7.5 Hz, 6H), 1.94–0.84 (m, 12H); ^13^C NMR (101 MHz, CDCl_3_) *δ* 150.2, 148.5, 147.5, 146.1, 145.5, 138.7, 138.0, 134.7, 133.8, 133.5, 132.1, 129.7, 129.7, 129.6, 127.7, 127.2, 126.6, 126.4, 126.1, 125.4, 124.7, 123.6, 123.5, 122.7, 118.4, 116.1, 116.0, 51.3, 36.1, 30.8, 28.7, 24.1, 23.2, 18.6, 14.2, 14.1, 11.8, 10.6; *m*/*z* (FAB^+^) 1189.6061 [M^+^] (C_77_H_82_^11^BF_2_N_5_S_2_ requires 1189.6078).

### Synthesis of PTZ-TPA-BDP

Compounds 8 (0.10 g, 0.16 mmol) and 6 (0.22 g, 0.82 mmol) were dissolved in dry toluene (15 mL) containing activated molecular sieves (10 mg, 4 Å). Acetic acid (0.8 mL) and piperidine (0.8 mL) were added into the solution and the mixture was stirred under an argon atmosphere under reflux until consumption of the starting material was confirmed by TLC. The mixture was cooled to room temperature and the solvent was evaporated under reduced pressure. Subsequently, the reaction mixture was washed with brine (50 mL), extracted with DCM (3 × 50 mL), dried over MgSO_4_ and filtered. After evaporation of the solvent under reduced pressure, flash column chromatography was performed over silica gel (CHCl_3_ : petroleum ether; 1 : 1) to yield the desired product PTZ-TPA-BDP as a dark green solid (0.040 g, 21%). Mp: 247–249 °C; ^1^H NMR (400 MHz, CDCl_3_) *δ* 7.67 (d, *J* = 16.7 Hz, 2H), 7.48 (d, *J* = 8.7 Hz, 4H), 7.33–7.26 (m, 7H), 7.24–7.10 (m, 13H), 7.09–7.04 (m, 10H), 7.00–6.90 (m, 3H), 3.80 (d, *J* = 7.1 Hz, 2H), 2.61 (q, *J* = 7.2 Hz, 4H), 2.03–1.94 (m, 1H), 1.51–1.38 (m, 10H), 1.31–1.26 (m, 4H), 1.16 (t, *J* = 7.5 Hz, 6H), 0.92–0.86 (m, 6H); ^13^C NMR (101 MHz, CDCl_3_) *δ* 150.4, 148.3, 147.4, 146.0, 145.7, 138.6, 136.8, 135.3, 133.8, 133.4, 133.3, 131.6, 129.8, 129.4, 128.4, 127.7, 127.6, 127.4, 127.3, 125.3, 124.9, 123.4, 123.1, 122.8, 118.5, 116.5, 116.1, 51.4, 36.0, 30.8, 28.7, 24.1, 23.2, 18.5, 14.2, 12.0, 12.0, 10.7; *m*/*z* (FAB^+^) 1123.5527 [M^+^] (C_75_H_72_^11^BF_2_N_5_S requires 1123.5575).

### Synthesis of N-PTZ-BDP

Compounds 10 (0.30 g, 0.71 mmol) and 4 (1.20 g, 7.31 mmol) were dissolved in dry toluene (20 mL) containing activated molecular sieves (30 mg, 4 Å). Acetic acid (3.5 mL) and piperidine (3.5 mL) were added into the solution and the mixture was stirred under an argon atmosphere under reflux until consumption of the starting material was confirmed by TLC. The mixture was cooled to room temperature and the solvent was evaporated under reduced pressure. Subsequently, the reaction mixture was washed with brine (50 mL), extracted with DCM (3 × 50 mL), dried over MgSO_4_ and filtered. After evaporation of the solvent under reduced pressure, flash column chromatography was performed over silica gel (CHCl_3_ : petroleum ether; 1 : 1) to yield the desired product N-PTZ-BDP as a dark green solid (0.211 g, 28%). Mp: 254–256 °C; ^1^H NMR (400 MHz, CDCl_3_) *δ* 8.39 (d, *J* = 8.7 Hz, 2H), 7.65 (d, *J* = 16.7 Hz, 2H), 7.55 (d, *J* = 8.7 Hz, 2H), 7.49 (dd, *J* = 8.5, 1.9 Hz, 2H), 7.39 (d, *J* = 1.9 Hz, 2H), 7.25–7.12 (m, 6H), 6.99–6.86 (m, 6H), 3.78 (d, *J* = 7.1 Hz, 4H), 2.59 (q, *J* = 7.4 Hz, 4H), 2.05–1.91 (m, 2H), 1.53–1.37 (m, 8H), 1.34–1.23 (m, 14H), 1.15 (t, *J* = 7.5 Hz, 6H), 0.89 (m, *J* = 7.3 Hz, 12H); ^13^C NMR (101 MHz, CDCl_3_) *δ* 151.2, 148.3, 146.5, 145.3, 143.5, 137.8, 135.6, 134.5, 134.1, 132.4, 131.9, 131.0, 130.5, 127.7, 127.3, 126.7, 126.2, 125.3, 124.3, 122.8, 118.0, 116.1, 116.1, 51.3, 36.1, 30.8, 28.7, 24.1, 23.2, 18.5, 14.1, 14.1, 12.0, 10.6; *m*/*z* (FAB^+^) 1067.5175 [M^+^] (C_65_H_72_^11^BF_2_N_5_O_2_S_2_ requires 1067.5194).

### UV-vis spectroscopy

Solution UV-vis spectra were recorded using a PerkinElmer Lambda 25 UV/VIS Spectrometer. Solutions for spin-coating films were made by dissolving 10 mg mL^−1^ of the relevant BODIPY molecule in chloroform. The solution was then spin-coated onto fused silica substrates at 800 rpm for 60 s. The UV-vis spectra of thin films were recorded with a CARY 300 UV-vis spectrophotometer and the thicknesses of the films were estimated using a Bruker Dektak 150 profilometer.

### Photoluminescence spectroscopy

Photoluminescence quantum yield (PLQY) of the neat donor films was measured using an integrating sphere based measurement system (Hamamatsu, C9920-02) under nitrogen flow.^37^ The films were excited at a wavelength of 350 nm. Fluorescence spectra and decay kinetics of neat films and blends with PC_71_BM were measured with a Hamamatsu streak camera coupled to a spectrograph. Excitation was by 200 fs laser pulses at 100 kHz repetition rate from an optical parametric amplifier pumped by a Light Conversion Pharos regenerative amplifier. Films were spin-coated and measured in nitrogen atmosphere. The spectra were corrected for the wavelength-dependent sensitivity.

### Electrochemistry

Solution electrochemistry (CV and SWV) was undertaken using a CH Instrument Electrochemical Workstation (CHI 440a), Austin, TX, USA. The supporting electrolyte was TBA[PF]_6_ (0.1 M in DCM) and samples were recorded at a concentration of 5 × 10^−4^ M and a scan rate of 0.07 V s^−1^. A platinum disk working electrode, a platinum wire counter electrode and a silver wire pseudo reference electrode were used for all measurements. The redox couples are referenced to ferrocene (external reference) with the Fc/Fc^+^ (*E*_1/2ox_ = 0.35 eV) redox couple adjusted to 0.0 V. Ionisation potentials (IP), electron affinities (EA) and *E*_fund_ were calculated using:

1 IP = −[*E*_ox_ + 4.8] eV

2 EA = −[*E*_red_ + 4.8] eV

3 *E*_fund_ = |IP − EA| eV

### DFT modelling

DFT calculations were undertaken using the Gaussian 09 software suite.^38^ Molecular geometries were initially optimized semi-empirically (PM6) and then re-optimized by DFT B3LYP/6-31G(d,p) level. The 2-ethylhexyl chains were replaced by methyl units to facilitate the convergence of the geometry optimizations. Minimum energy structures were confirmed by the absence of imaginary frequencies in the calculated IR spectra.

### Solar cell fabrication and characterisation

Organic solar cells were fabricated on pre-patterned ITO-coated glass. The ITO-coated glass substrates were cleaned in detergent (sodium dodecyl sulphate), successively ultrasonicated in deionized water, acetone, and isopropyl alcohol, and exposed to oxygen in a plasma asher for 3 minutes. For each donor molecule, the photovoltaic performance optimization process was started with identifying the donor to acceptor ratio (wt%, varying from 1 : 1 to 1 : 6) giving the best photovoltaic performance. Then thermal annealing process (room temperature to 150 °C) was applied to maximize the OPV device performance. The acceptor molecule used was PC_71_BM (American Dye Sources, ADS71BFA) and the total concentration of the D:A blend mixture was 10 mg mL^−1^ in chloroform. Donor and acceptor solutions were stirred separately for ∼6 hours and then the blended mixture was stirred for another 3 hours. Devices were made by depositing PEDOT:PSS as hole injection layer having thickness ∼ 30–40 nm. The active layer was deposited by spin - coating (800 rpm, 60 s) on glass/ITO/PEDOT:PSS substrates inside a nitrogen filled glove box. The samples were then transferred to a vacuum thermal evaporator (1 × 10^−6^ mbar base pressure) and kept under vacuum before thermally evaporating the metal contacts consisting of Ca (5 nm) and Al (100 nm) using a shadow mask. The active area of the device was 0.08 cm^2^. All the processing related to the active layer was performed inside the nitrogen glove box.

After the electrode deposition, the devices were encapsulated with a UV optical adhesive and a glass coverslip. The current–voltage characteristics were determined under an illumination intensity of 100 mW cm^−2^ in air using an air mass 1.5 global (AM 1.5G) Sciencetech solar simulator and a Keithley 2400 source-measure unit. The illumination intensity was verified with a calibrated monosilicon detector with a KG-5 filter. The external quantum efficiency (EQE) measurements were performed at zero bias by illuminating the device with monochromatic light supplied from a xenon arc lamp in combination with a dual-grating monochromator.

## Results and discussion

### Synthesis

**Scheme 1:**
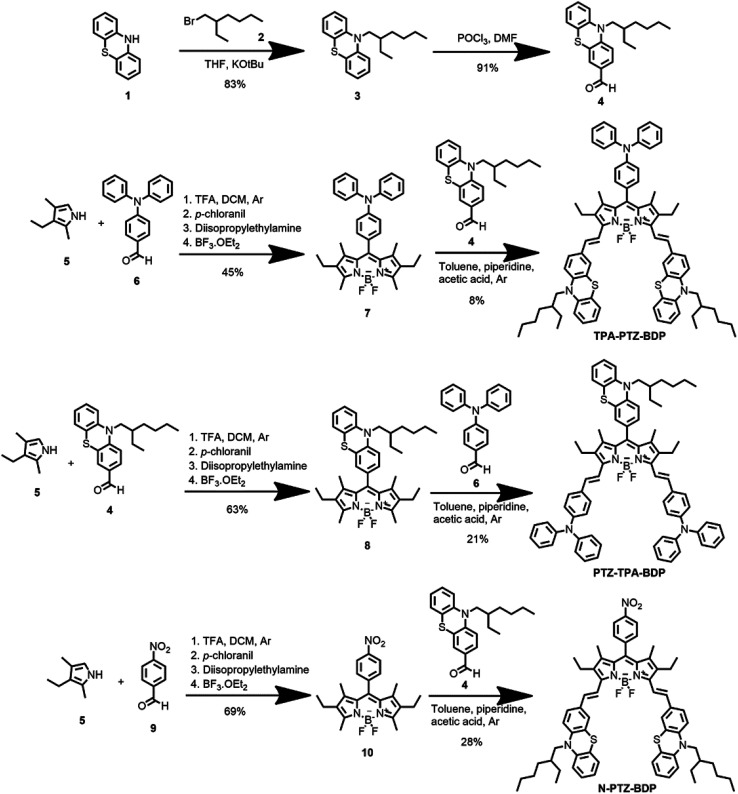
Synthesis of compounds TPA-PTZ-BDP, PTZ-TPA-BDP and N-PTZ-BDP.

The synthesis of BODIPYs TPA-PTZ-BDP, PTZ-TPA-BD, and N-PTZ-BDP involved the multistep approach shown in Scheme 1. PTZ aldehyde 4 was synthesized following a slightly modified procedure from the literature, which consisted of alkylation of 10*H*-phenothiazine followed by a Vilsmeier formylation protocol.^36,39^ The BODIPY derivatives were synthesized through well-established protocols,^40–42^ that follows a Knoevenagel type condensation as final step to obtain the target compounds with yields shown in Scheme 1.

### Optical properties

**Fig. 2:**
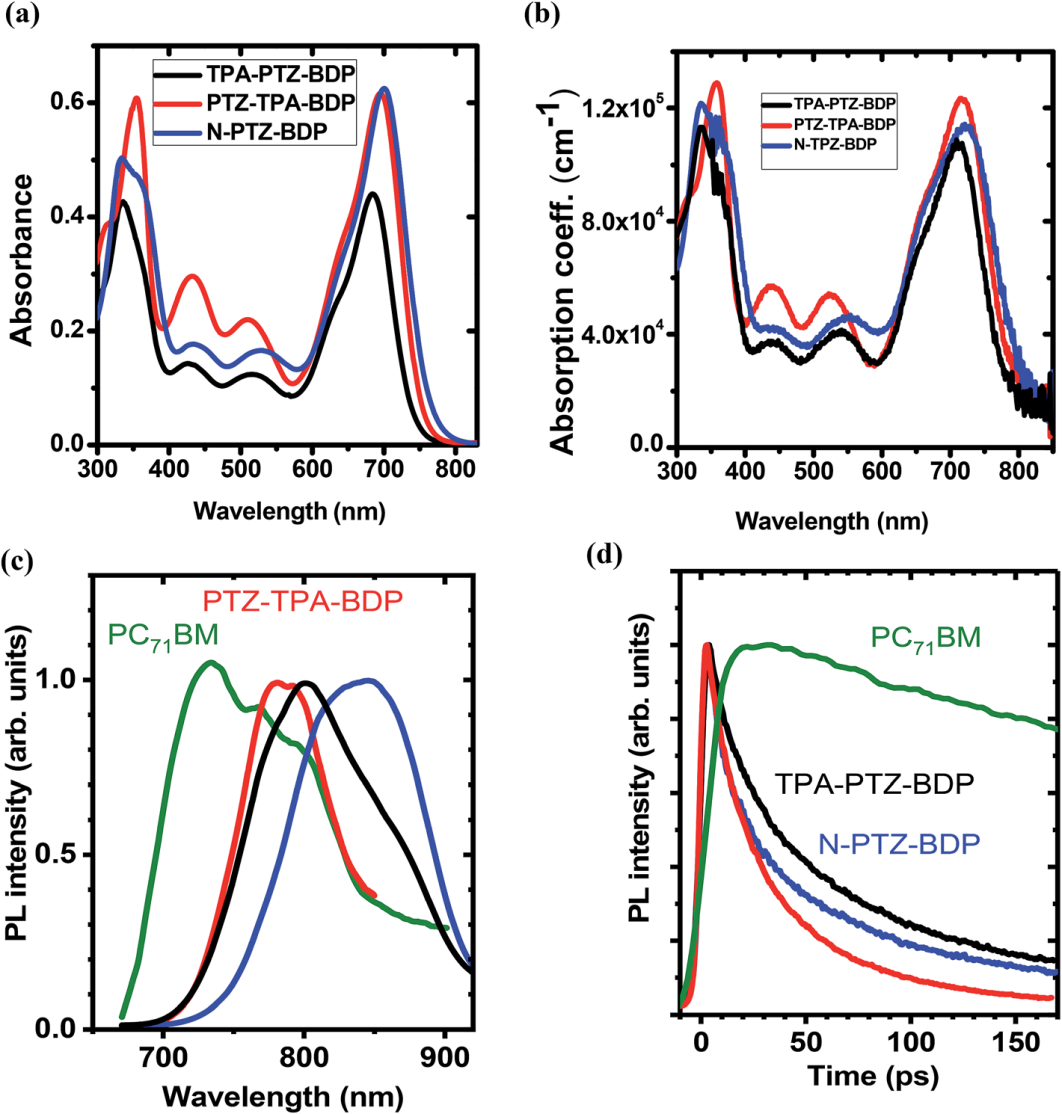
(a) Absorption spectra of TPA-PTZ-BDP, PTZ-TPA-BDP and N-PTZ-BDP recorded in DCM solution (1 × 10^−5^ M) and in (b) thin film form. (c) Normalised time-integrated fluorescence spectra of BODIPY derivatives (d) decay kinetics of the neat films recorded after excitation at 515 nm for PC_71_BM and at 660 nm for BODIPY derivatives. Kinetics were obtained by integrating fluorescence spectra over a 150 nm window for BODIPY derivatives and a 200 nm window for PC_71_BM. Because of the weak fluorescence emission of BODIPY derivatives, the plots shown in (c) are smoothed for clarity of spectral shape.

The UV-vis absorption spectra of compounds TPA-PTZ-BDP, PTZ-TPA-BDP and N-PTZ-BDP were recorded in DCM solution (1 × 10^−5^ M) and as thin films on fused silica discs. The spectra are shown in Fig. 2(a) and (b) and the optical data are summarized in Table 1. As can be seen in Fig. 2(a), the three dyes (in solution) show similar values of absorption maximum (*λ*_max_) in the visible range, 686 nm for TPA-PTZ-BDP, 697 nm for PTZ-TPA-BDP, and 702 nm for N-PTZ-BDP. The bands centered at around 340 nm are attributed to the π–π* transition originating from the conjugated backbone, whereas the bands centered at around 700 nm correspond to the S_0_–S_1_ transition due to the intramolecular charge transfer (ICT) between donor and acceptor moieties. The latter is expected to originate primarily from the donor moieties (located on the α-positions) to the electron-deficient core upon photoexcitation since the degree of conjugation between the donor unit located at the *meso*-position and the core is often low.^14,43^ The parent BODIPY dye molecule absorbs in the visible in the range 400–600 nm.^44,45^ Compounds 7, 8 and 10 show absorption maxima around 520 nm^40^ [the spectra of compound 8 is given in ESI, Fig. S9†], whereas TPA-PTZ-BDP, PTZ-TPA-BDP and N-PTZ-BDP show absorption maxima at around 700 nm. Therefore, we found that the introduction of PTZ groups in the α-positions leads to a bathochromic effect of circa 175 nm. Interestingly, N-PTZ-BDP shows a slight red-shifted spectrum in comparison with its counterparts. This suggests that the nitro group present in the *meso*-moiety increases the push–pull character of the molecule. This finding is interesting since this effect can increase the charge transfer capability of the molecule. The *ε*_max_ of the molecules were found to be 4.4 × 10^4^ cm^−1^ M^−1^ for TPA-PTZ-BDP, 6.2 × 10^4^ cm^−1^ M^−1^ for PTZ-TPA-BDP and 6.3 × 10^4^ cm^−1^ M^−1^ for N-PTZ-BDP. The optical band-gaps (*E*_opt_) were estimated according to the formula *E*_opt_ = 1240/*λ*_onset_, and the values were found as 1.64, 1.59 and 1.57 eV for TPA-PTZ-BDP, PTZ-TPA-BDP and N-PTZ-BDP, respectively (see Table 1). In summary, our molecular design strategy was successful in creating dyes with panchromatic absorption covering a large spectral range from 300 nm to 850 nm with high extinction coefficients, which is desirable for solar cell applications.

**Table 1:** Summary of the optical and the electrochemical data of compounds TPA-PTZ-BDP, PTZ-TPA-BDP and N-PTZ-BDP

Dye	*λ* a (nm)	*λ*_max_ b (nm)	*ε*_max_	*λ*_onset_ (nm)	*E*_opt_ (eV)	*E*_1/2ox_ (V)	*E*_1/2red_ (V)	IP (eV)	EA (eV)	*E*_fund_ (eV)
TPA-PTZ-BDP	335	711	4.4 × 10^4^	754	1.64	0.23	−1.40	−5.03	−3.40	1.63
	686									
PTZ-TPA-BDP	355	717	6.2 × 10^4^	777	1.59	0.20	−1.38	−5.00	−3.42	1.58
	697									
N-PTZ-BDP	334	721	6.3 × 10^4^	786	1.57	0.26	−1.30	−5.06	−3.50	1.56
	702									

a Measured in DCM solution.

b Measured in thin film.

The absorption spectra of thin films of TPA-PTZ-BDP, PTZ-TPA-BDP and N-PTZ-BDP are shown in Fig. 2(b) and show a similar profile to those in solution. However, they show a bathochromic shift of circa 20–25 nm and slightly broader absorption bands which is in common with thin films of conjugated materials.^43,46^ The absorption coefficient of the molecules in thin films were found to range between 1 × 10^5^–1.2 × 10^5^ cm^−1^ at the peak absorption wavelength of ∼700 nm. The optical bandgap estimated from the absorption onset in thin film forms are 1.58, 1.56 and 1.53 eV for TPA-PTZ-BDP, PTZ-TPA-BDP and N-PTZ-BDP, respectively and hence close to the optimal value of band gap energy required for a single junction solar cells.^47,48^

The PL emission spectra of the neat donor and acceptor films are shown in Fig. 2(c). The fluorescence spectra of the three BODIPY derivatives show broad maxima around 800 nm. The measured fluorescence of all the films was very weak and below the 1% threshold sensitivity of our PLQY measurement. The PL decay kinetics shown in Fig. 2(d) shows that the fluorescence lifetime of the BODIPY derivatives is rather short (varies between 30–60 ps for 1/*e* decay), which is consistent with the weak fluorescence observed.

### Electrochemical properties

**Fig. 3:**
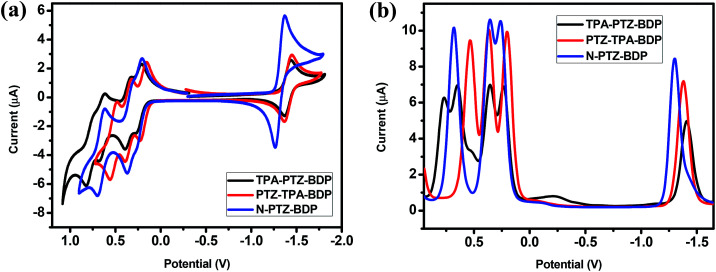
(a) Cyclic voltammetry (b) square wave voltammetry traces of TPA-PTZ-BDP, PTZ-TPA-BDP and N-PTZ-BDP, recorded in DCM (5 × 10^−4^ M), calibrated *versus* the ferrocene/ferrocenium (Fc/Fc^+^) redox couple, using TBA·PF_6_ as electrolyte, a 1.6 mm diameter platinum working electrode, a platinum wire counter electrode and a silver wire pseudo reference electrode.

The electrochemical behavior of the dyes was investigated by cyclic voltammetry (CV) and square wave voltammetry (SWV). Fig. 3 shows the onset of oxidation (*E*_ox_) and reduction (*E*_red_) of the compounds, where at least one reduction and three oxidation waves can be observed for each compound. It is interesting to notice the near-reversible character of the redox waves of these dyes from the CV data displayed in Fig. 3(a). This character is important for the stability of organic solar cells since the dye needs to regenerate upon every cycle. The more positive reduction potential of N-PTZ-BDP is likely a consequence of the push–pull nature of this compound resulting from the addition of the nitrobenzene moiety, whereas the slightly lower oxidation potential of TPA-PTZ-BDP and PTZ-TPA-BDP in comparison with N-PTZ-BDP is presumably due to the donating units attached to the *meso*-position of the BODIPY core. The ionization potential (IP), electron affinity (EA), and consequent fundamental gap (*E*_fund_) were estimated according to the eqn (1), (2) and (3), respectively. The electrochemical data and IP and EA values are summarized in Table 1. The IP values were obtained as 5.03 eV for TPA-PTZ-BDP, 5.00 eV for PTZ-TPA-BDP and 5.06 eV for N-TPA-BDP, and the EA values were obtained as 3.40 eV for TPA-PTZ-BDP, 3.42 eV for PTZ-TPA-BDP and 3.50 eV for N-TPA-BDP. Consequently, the *E*_fund_ of the molecules were found to be 1.63, 1.58 and 1.56 eV for TPA-PTZ-BDP, PTZ-TPA-BDP and N-TPA-BDP, respectively.

### Theoretical calculations

**Fig. 4:**
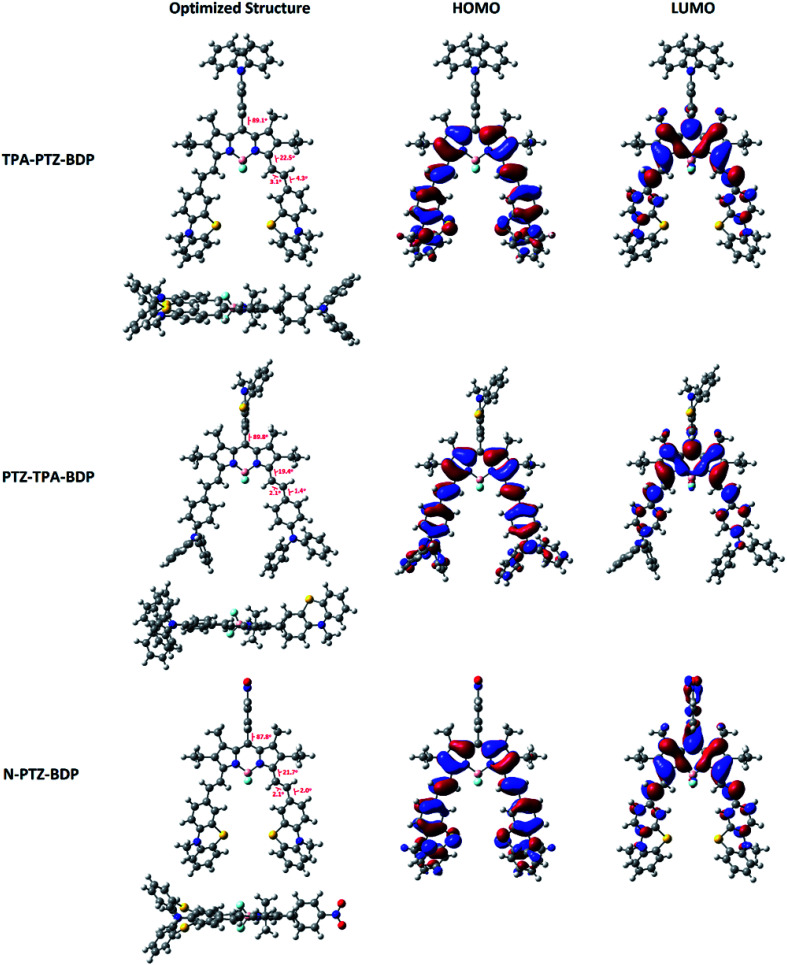
Frontier orbitals (HOMO and LUMO) of compounds TPA-PTZ-BDP, PTZ-TPA-BDP and N-PTZ-BDP computed at the B3LYP level of theory and the 6-31G(d,p) basis set. Molecular geometries were first optimized semi-empirically, and then re-optimized using DFT.

In order to understand more about the electronic properties of the dyes, density functional theory (DFT) calculations at the B3LYP/6-31G(d,p) level of theory were undertaken. Firstly, as can been seen in Fig. 4, the calculations confirmed that the moieties attached to the *meso*-position are perpendicular to the core in all molecules and that the dihedral angle between BODIPY and *meso*-units are 89.1° for TPA-PTZ-BDP, 89.8° for PTZ-TPA-BDP and 87.8° for N-PTZ-BDP. On the other hand, a higher degree of co-planarity between the styryl donor units with the BODIPY core was observed, demonstrating that the π-conjugation is efficient through the α-position for the three dyes. The highest occupied molecular orbital (HOMO) and the lowest unoccupied molecular orbital (LUMO) maps (see Fig. 4) clearly indicate that the HOMO is primarily located on the styryl groups extending to the BODIPY core for all three compounds, whereas the LUMO is confined to the π-framework of the BODIPY core extending only to the styryl groups in the case of TPA-PTZ-BDP and PTZ-TPA-BDP. Poor electronic communication observed between the *meso*-substituents and the core is usually associated with the presence of methyl groups in the positions 1- and 7-, which leads to the orthogonal arrangement. However, the LUMO of N-PTZ-BDP is interestingly located on both the nitrobenzene group attached in the *meso*-position and the BODIPY core extending to the styryl groups. This finding is interesting and corroborates with the suggestion that the nitrobenzene moiety might increase the push–pull character of the molecule. Therefore, in N-PTZ-BDP the ICT effect should be more pronounced than in the other dyes.

### Photovoltaic properties

**Fig. 5:**
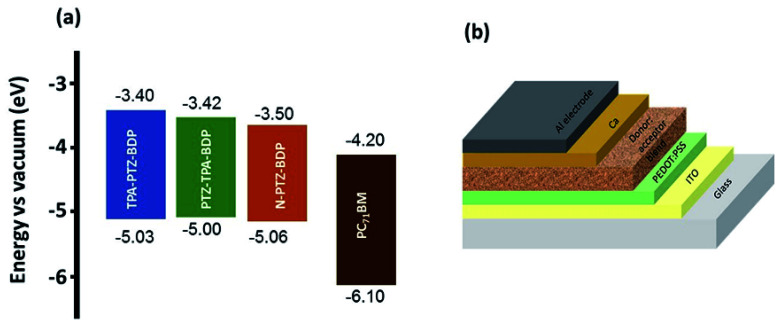
(a) Energy level diagram of TPA-PTZ-BDP, PTZ-TPA-BDP, N-PTZ-BDP and PC_71_BM (b) solar cell device architecture used to fabricate OPVs in this study.

Solution processed BHJ devices were fabricated using TPA-PTZ-BDP, PTZ-TPA-BDP and N-PTZ-BDP as the electron donor material and PC_71_BM as the acceptor material. The energy level diagram of the donor and acceptor molecules are shown in Fig. 5(a) and the device architecture (glass/ITO/PEDOT:PSS/active layer/Ca/Al) used for fabricating OPVs is shown in Fig. 5(b). The photovoltaic performance of the dyes were evaluated systematically with careful optimization of the processing parameters such as ratio of donor to acceptor (D : A) and thermal annealing temperature.

**Fig. 6:**
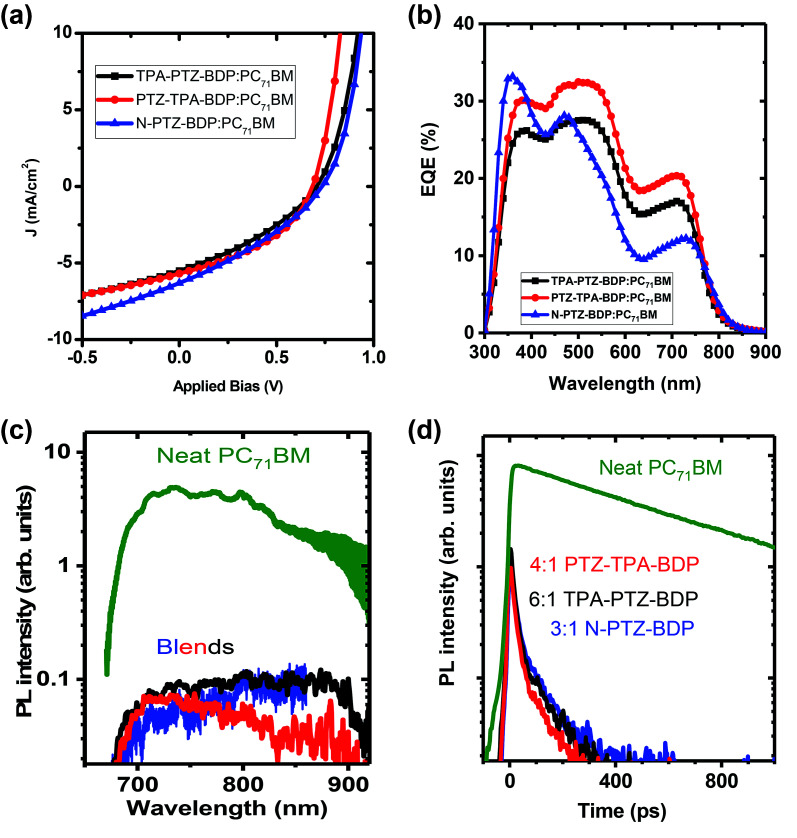
(a) The *J*–*V* characteristics of the best performing OPVs for TPA-PTZ-BDP, PTZ-TPA-BDP and N-PTZ-BDP molecules for their optimized blend ratio with PC_71_BM. (b) EQE spectra of the above devices. (c) Steady state fluorescence spectra and (d) decay kinetics of a neat PC_71_BM film and of the optimised photovoltaic blends containing the PC_71_BM : BODIPY ratio shown, recorded after excitation at 515 nm and normalised to the absorbed excitation power. Kinetics were obtained by integrating fluorescence spectra from 680–850 nm. The vertical scale is logarithmic.

In the case of small molecule donor based OPVs, the relative content of the donor and acceptor molecule is crucial in obtaining a homogeneously mixed BHJ morphology for efficient exciton harvesting and for the formation of sufficient charge transport network.^7,49^ The optimized weight ratio of each of the donor molecules to PC_71_BM is obtained by varying it from 1 : 1 to 1 : 6 and keeping the total concentration 10 mg mL^−1^. For TPA-PTZ-BDP, PTZ-TPA-BDP and N-PTZ-BDP these optimized donor to acceptor ratios are respectively 1 : 6, 1 : 4 and 1 : 3. Under these optimized D : A weight ratio, TPA-PTZ-BDP:PC_71_BM blend shows a PCE_max_ of 1.33%, PTZ-TPA-BDP:PC_71_BM has a PCE_max_ of 1.62%, and N-PTZ-BDP:PC_71_BM shows a PCE_max_ of 1.51%. The photovoltaic performance parameters, the corresponding *J*–*V* characteristics and the EQE spectra of the best OPVs under optimized D : A ratio for each of the BODIPY dye molecule are shown in Fig. 6(a) and (b) and Table 2. The details of the D : A wt% optimization details are given in ESI (Fig. S10(a)–(c), S11(a), (b) and Table S3–S5.† For all three BODIPY molecules, following an increase in fullerene content, the short circuit current density (*J*_sc_) (and hence the EQE (Fig. S11(a) and (b)†) increases systematically but with relatively smaller effects observed on open circuit voltage (*V*_oc_) and fill factor (FF). It has been previously reported that higher content of fullerene molecules in the oligothiophene:fullerene blends prevents the crystallization of oligothiophenes and enables the formation of a desirable carrier transport network with finer mixing of D : A components.^49,50^ This finer mixing and better carrier transport pathways can increase the exciton harvesting and charge collection, thus accounting for the improved short circuit current density with higher PC_71_BM content. Improvement in charge transport pathways can also account for the slightly improved FF with increase in fullerene content in the blend. In contrast, *V*_oc_ decreases slightly with increasing acceptor content. This can be due to the finer morphology of the BHJ blend with increasing content of the fullerene and/or the tail states near the quasi Fermi level due to the amorphous nature of the fullerene.^51,52^ It is noteworthy that although these donor molecules possess high absorption coefficients in the near infrared region (Fig. 2(b)), the *J*_sc_ values of the OPV devices are rather low ∼5–6 mA cm^−2^. This low short circuit current density could be due to unfavorable morphology of the donor–acceptor blend (*vide infra*) which can limit the photogenerated exciton dissociation or enhance the recombination losses of the photogenerated charge carriers. This is further discussed in detail below. Furthermore, the *V*_oc_ of these OPVs are in the range of 0.6–0.7 V, which is due to the relatively shallow HOMO levels of these donor molecules as shown in Fig. 5(a).

**Table 2:** The OPV performance parameters (averaged over 8 devices) for different BODIPY molecules under optimized D : A wt ratio. The ± numbers refers to the standard deviation of each of the photovoltaic performance parameters over the 8 devices. The *J*_sc_ estimated from EQE spectra is also included

Donor:acceptor blend	Donor to acceptor ratio	*J*_sc_ (mA cm^−2^) (*J*_sc_ from EQE)	*V*_oc_ (V)	FF (%)	PCE avg. (%)	PCE best (%)
TPA-PTZ-BDP:PC_71_BM	1 : 6	5.09 ± 0.36 (5.22)	0.687 ± 0.015	32.5 ± 1.1	1.14 ± 0.11	1.33
PTZ-TPA-BDP:PC_71_BM	1 : 4	5.70 ± 0.11 (6.20)	0.672 ± 0.008	40.8 ± 0.8	1.56 ± 0.04	1.62
N-PTZ-BDP:PC_71_BM	1 : 3	5.83 ± 0.44 (4.83)	0.699 ± 0.114	33.4 ± 6.7	1.33 ± 0.14	1.51

To further understand the kinetics of charge pair generation, and charge transfer processes, steady state and time resolved PL measurements were carried out for the optimised blend films of BODIPY and PC_71_BM. Fig. 6(c) and (d) shows fluorescence spectra and kinetics which were normalised to the absorbed excitation power at the excitation wavelength, hence, charge generation efficiency in the blends can be deduced from fluorescence intensity. Photovoltaic blends of PC_71_BM with BODIPY derivatives show similar spectra, albeit about 50 times weaker than the neat PC_71_BM film, which indicates that about 98% of excitons are quenched in the blends. Time-resolved fluorescence of the neat PC_71_BM film shows a mono-exponential decay with 600 ps lifetime. The blends show about five times weaker initial intensity than the neat PC_71_BM film suggesting that about 80% of charges are generated within the response function of the streak camera (∼10 ps in this case). Rapid charge generation implies intimate mixing of two materials in the blends. Such a morphology gives fast charge pair generation at the interface between donor and acceptor, but enhances the recombination losses as carrier transport is interrupted by domain boundaries.^53^ This leads to needing a significant electric field to dissociate charge pairs and extract them, leading to the low fill factors we observe here.

Compared to the blends of TPA-PTZ-BDP and PTZ-TPA-BDP with PC_71_BM, the N-PTZ-BDP:PC_71_BM blend shows higher open circuit voltage, (*V*_oc_) as shown in Fig. 6(a) and Table 2 and that can be related to its deeper HOMO level. The EQE spectra of the TPA-PTZ-BDP:PC_71_BM and PTZ-TPA-BDP:PC_71_BM follows a similar spectral profile, however N-PTZ-BDP:PC_71_BM blend shows a slightly different spectral shape with more absorption in the blue wavelength range. The absorption spectra of the corresponding blend films of N-PTZ-BDP:PC_71_BM at the optimised fullerene content is given in Fig. S12.†

**Fig. 7:**
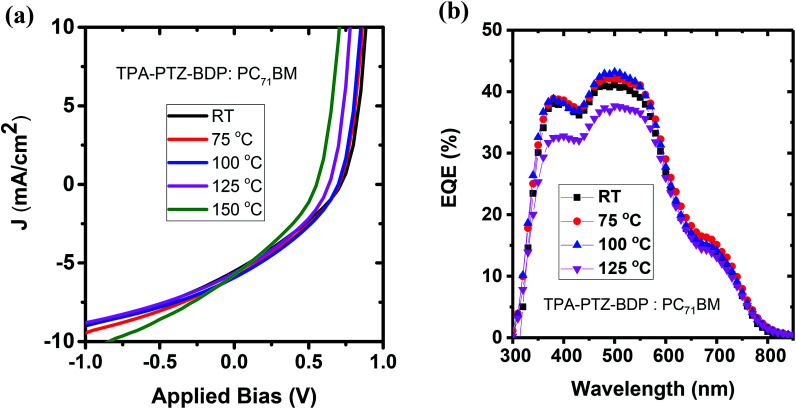
(a) *J*–*V* characteristics and (b) EQE spectra of TPA-PTZ-BDP:PC_71_BM as a function of different thermal annealing temperatures.

**Fig. 8:**
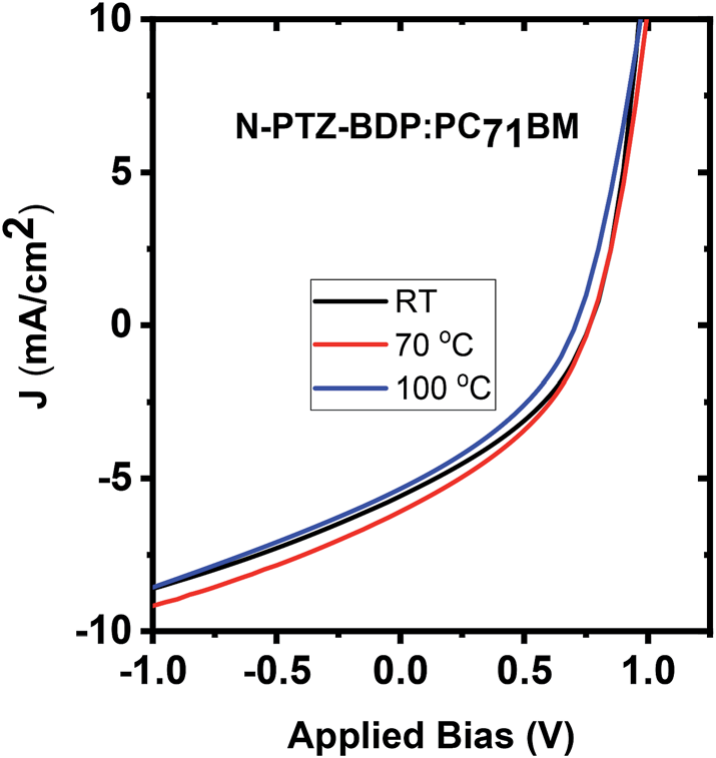
*J*–*V* characteristics of N-PTZ-BDP:PC_71_BM blend as a function of thermal annealing.

To investigate whether thermal annealing process can further improve the photovoltaic properties, under each optimized D : A weight ratio, the blends were thermally annealed at different temperatures up to 150 °C. In the case of TPA-PTZ-BDP:PC_71_BM blend, thermal annealing did not increase the PCE of the blend. The corresponding photovoltaic performance parameters as a function of thermal annealing are shown in Fig. 7(a) and Table 3. Similar thermal annealing also did not increase the performance of the PTZ-TPA-BDP:PC_71_BM blend. However for N-PTZ-BDP:PC_71_BM blend, thermal annealing at 70 °C improved the PCE from 1.33% to 1.71%. The corresponding *J*–*V* characteristics and photovoltaic performance parameters are listed in Fig. 8 and Table 4.

**Table 3:** Photovoltaic performance parameters of the TPA-PTZ-BDP:PC_71_BM blend as a function of different thermal annealing temperature. The ± numbers refers to the standard deviation of each of the photovoltaic performance parameters over the 8 devices

Anneal. temp. (°C)	*J*_sc_ (mA cm^−2^)	*V*_oc_ (V)	FF (%)	*R*_sh_ (ohmcm^2^)	*R*_s_ (ohmcm^2^)	PCE avg. (%)	PCE best (%)
RT	5.44 ± 0.20	0.686 ± 0.018	30.7 ± 0.4	186 ± 5	2.55 ± 1.05	1.15 ± 0.06	1.24
75	5.63 ± 0.28	0.685 ± 0.013	31.8 ± 0.5	190 ± 11	2.67 ± 1.20	1.23 ± 0.05	1.34
100	5.83 ± 0.11	0.671 ± 0.019	33.1 ± 0.5	202 ± 6	2.78 ± 1.16	1.30 ± 0.05	1.39
125	5.48 ± 0.11	0.622 ± 0.011	33.5 ± 0.4	197 ± 10	3.15 ± 0.83	1.15 ± 0.04	1.23
150	5.98 ± 0.32	0.553 ± 0.002	32.5 ± 0.8	140 ± 13	2.72 ± 0.50	1.08 ± 0.08	1.19

**Table 4:** Photovoltaic performance parameters of N-PTZ-BDP:PC_71_BM as a function of thermal annealing

Thermal annealing temperature (°C)	*J*_sc_ (mA cm^−2^)	*V*_oc_ (V)	FF (%)	PCE (%) avg.	PCE best (%)
RT	5.2 ± 0.4	0.762 ± 0.006	36.8 ± 0.5	1.46 ± 0.10	1.56
70	5.57 ± 0.54	0.768 ± 0.007	36.0 ± 1.3	1.54 ± 0.20	1.71
125	4.76 ± 0.54	0.694 ± 0.018	33.3 ± 2.7	1.11 ± 0.20	1.34

### Surface morphology by atomic force microscopy (AFM)

**Fig. 9:**
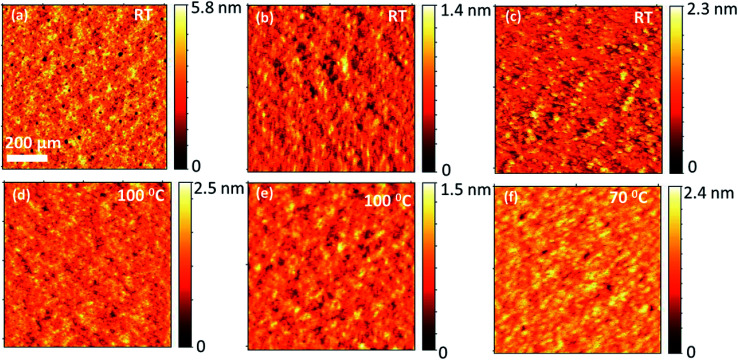
Comparison of surface morphology (height images) of optimized blend ratio of each of the BODIPY donor molecules with PC_71_BM for as-cast and annealed films; (a) & (d) for PTZ:TPA-BDP:PC_71_BM, (b) & (e) for TPA-PTZ-BDP:PC_71_BM (c) & (f) for N-PTZ:BDP:PC_71_BM.

To investigate how the three different BODIPY donor molecules form distinctive bulk heterojunction with PC_71_BM, the morphological properties of the blends were characterized using atomic force microscopy (AFM). The AFM images corresponding to the blends of TPA-PTZ-BDP:PC_71_BM, PTZ-TPA-BDP:PC_71_BM, and N-PTZ-BDP:PC_71_BM at their optimized D to A ratio are shown in Fig. 9(a)–(c). Very small domains implying fine mixing of the donor and acceptor components are seen from these AFM height images with root mean square surface roughness of less than 1 nm for all these blends. Thus as mentioned in the photovoltaic characterization section, at higher D : A blend ratio, (which is the optimum for three donors), this finer surface morphology can increase the photogeneration of charge carriers, but can increase the recombination losses which is reflected in the slightly reduced open circuit voltages at higher acceptor content (Table S3–S5†). Moreover, this finer morphology of the blends, lacking bi-continuous percolative pathways to the charge collecting electrodes could be the reason for the low short circuit density and the low FF, as the possibility of charge recombination is higher for this kind of finely mixed donor and acceptor blend.^54–56^ Compared to TPA-PTZ-BDP:PC_71_BM and PTZ-TPA-BDP:PC_71_BM blends, N-PTZ-BDP:PC_71_BM (Fig. 9(c)) shows slightly larger domain size for the bulk heterojunction morphology. With thermal annealing, only a marginal increase in domain size is observed for TPA-PTZ-BDP:PC_71_BM and PTZ-TPA-BDP:PC_71_BM blends [Fig. 9(d) and (e)] whereas for N-PTZ-BDP:PC_71_BM blend (Fig. 9(f)), well defined granular features and clearly larger domain size is observed. This optimized morphology may be the reason why the N-PTZ-BDP:PC_71_BM blend has slightly increased PCE with increase in thermal annealing (Table 4).

Thus our molecular engineering of BODIPY dye molecules by incorporating TPA and PTZ moieties and nitrobenzene in the *meso* and α-positions resulted in favourable optoelectronic properties for photovoltaic applications, such as panchromatic absorption and reversible electrochemical properties. However, their bulk heterojunction solar cells, fabricated by blending with PC_71_BM fullerenes resulted in limited photovoltaic power conversion efficiencies which could be due to the poor charge transport properties of new BODIPY dyes and suggest further work is needed to improve charge extraction.

## Conclusions

Three new BODIPY donor materials featuring PTZ moieties as donor units at the α-positions or as *meso*-linker, TPA-PTZ-BDP, PTZ-TPA-BDP and N-PTZ-BDP, were designed and synthesized. In N-PTZ-BDP, the introduction of an electron withdrawing motif (nitro) in the *meso*-substituent appears to have a beneficial effect on the mechanism of charge transfer. We found that the three dyes possess excellent panchromatic absorption covering the spectra from 300 nm to 850 nm with high extinction coefficient. However, N-PTZ-BDP showed a more extended HOMO–LUMO overlay profile revealed by DFT, and a slightly red-shifted spectra in both solution and film. The small molecules showed compatible photoelectric characteristics for the application as donor in BHJ solar cells together with PCB_71_M. With respect to the photovoltaic performance comparison, PTZ-TPA-BDP showed the highest PCE of 1.62% under the optimized D : A ratio whereas with thermal annealing the photovoltaic properties of N-PTZ-BDP:PC_71_BM blends showed the highest PCE of 1.71%. Therefore, as a result of the overall molecular composition, N-PTZ-BDP exhibited the highest conversion efficiency among the three dyes after optimization of the BHJ. This can be due to the larger length scale of phase separation and well-defined granular features observed in the BHJ morphology by AFM analysis. Hence, it is sensible to conclude that introducing the electronegative moiety on the *meso*-position had a positive effect on the absorption spectra of the BODIPY derivative extending it further towards the near infrared. Also, the near-reversible character of the redox waves of these dyes are important features that account for device stability. Finally, the findings discussed in this contribution show that the PTZ groups can make effective electron donor moieties as vinyl-substituents at the α-position with a high degree of planarity leading to dyes that possess panchromatic absorption, and indicate that the introduction of acceptor moieties in the *meso*-position is promising for high performance BODIPY-based BHJ solar cells.

## Conflicts of interest

No conflicts to declare.

## Supplementary Material

RA-009-C9RA01750J-s001
