# Supplementary material for: BODIPY derivatives with near infra-red absorption as small molecule donors for bulk heterojunction solar cells
Source: RSC Adv. 2019 May 16;9(27):15410–23. doi: 10.1039/c9ra01750j (PMC9064333; doi:10.1039/c9ra01750j)
Supplement: RA-009-C9RA01750J-s001 [file RA-009-C9RA01750J-s001.pdf]

Electronic Supplementary Material (ESI) for RSC Advances.
This journal is © The Royal Society of Chemistry 2019

Electronic Supporting Information

New BODIPY derivatives with near infra-red absorption as small molecule donors for bulk heterojunction solar cells

John Marques Dos Santos,[a¶] Lethy Krishnan Jagadamma,[b¶] Najwa Mousa Latif,[a] Arvydas Ruseckas,[b] Ifor D. W. Samuel,[b*] Graeme Cooke[a*]

[a.] *WestCHEM, School of Chemistry, University of Glasgow, Glasgow, G12 8QQ, UK.*

[b.] *Organic Semiconductor Centre, SUPA, School of Physics and Astronomy, University of St. Andrews, St. Andrews, Fife, KY16 9SS, UK.*

E-mail: Graeme.Cooke@glasgow.ac.uk; idws@st-andrews.ac.uk

ESI1 Materials.

All the reagents were purchased from Sigma Aldrich®, Fluorochem®, TCI®, Alfa Aesar®, Acros® or Fisher Scientific® and used as received. Column chromatography was carried out using silica gel (Sigma-Aldrich) 40 – 63 µm particle size, 60 Å pore size. The solvent system is specified in each experiment. Thin-layer chromatography (TLC) was performed using Merck silica gel 60 covered aluminium plates F254. Dry solvents were obtained from Innovative Technology inc. Pure Solv 400-5-MD solvent purification system (activated alumina columns) or Sigma Aldrich®.

ESI2 NMR Spectra.

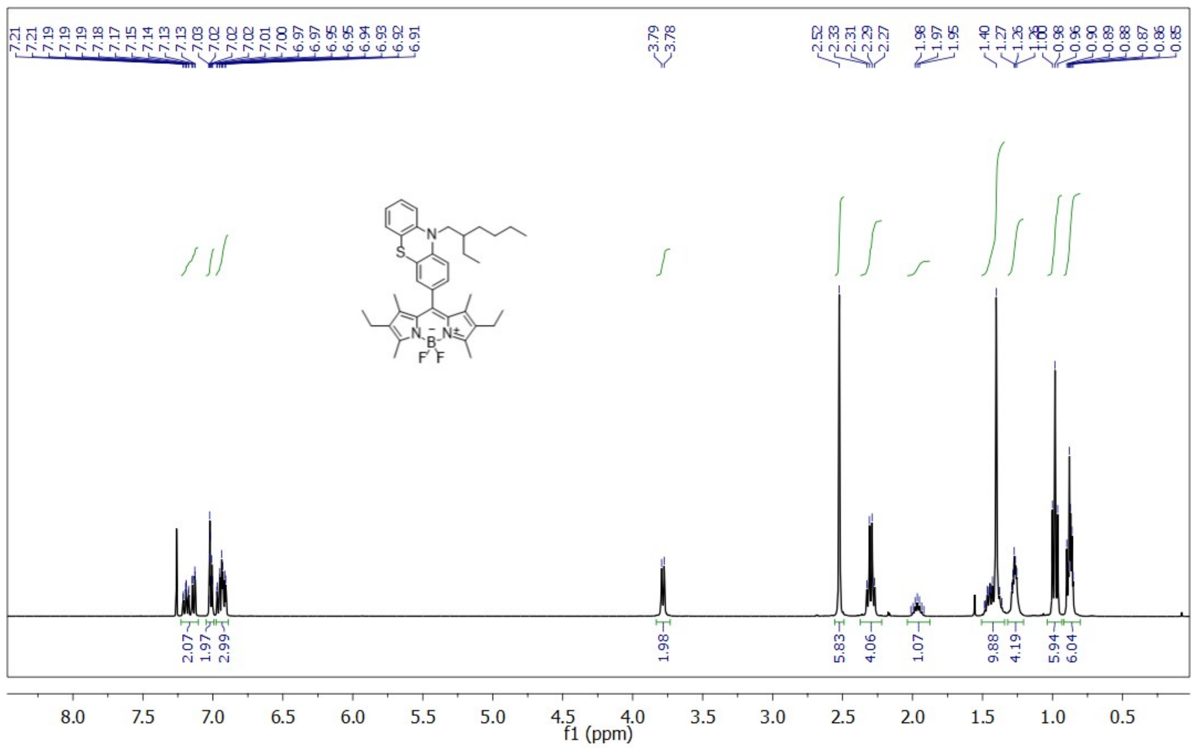

Figure S1: ^{1}H NMR (400 MHz, $CDCl_3$) of compound **8**.

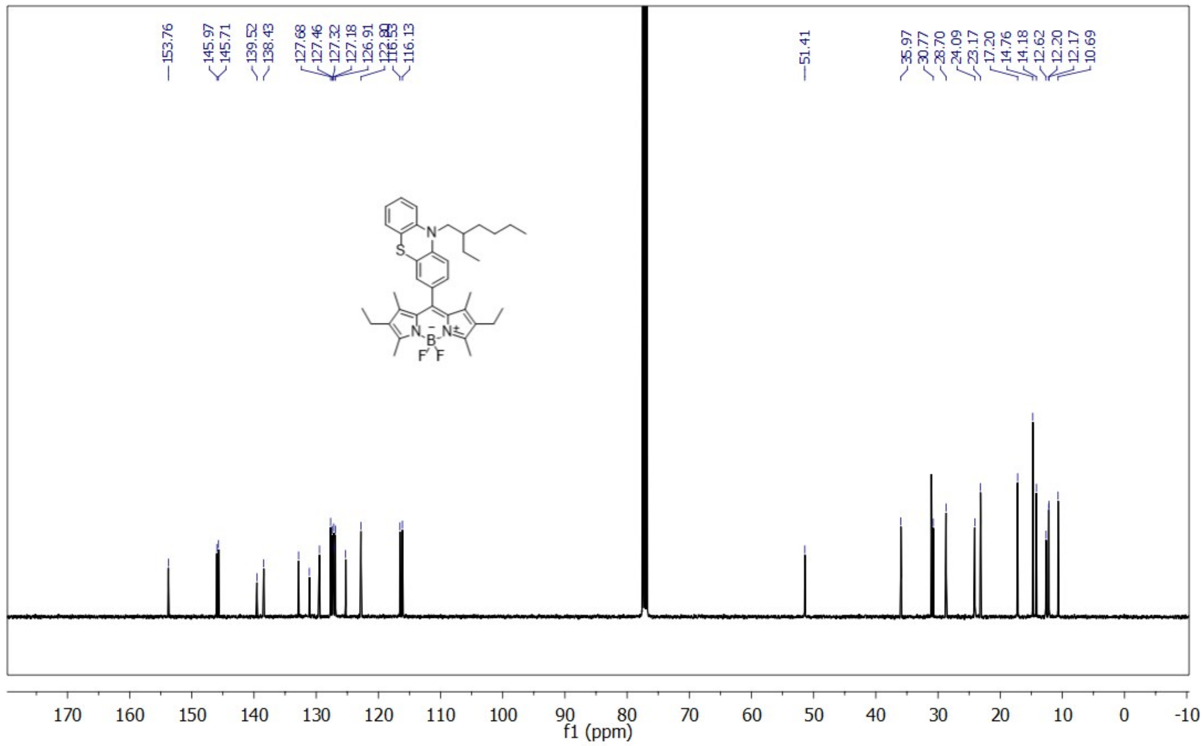

Figure S2. ^{13}C NMR (100 MHz, $CDCl_3$) of compound **8**.

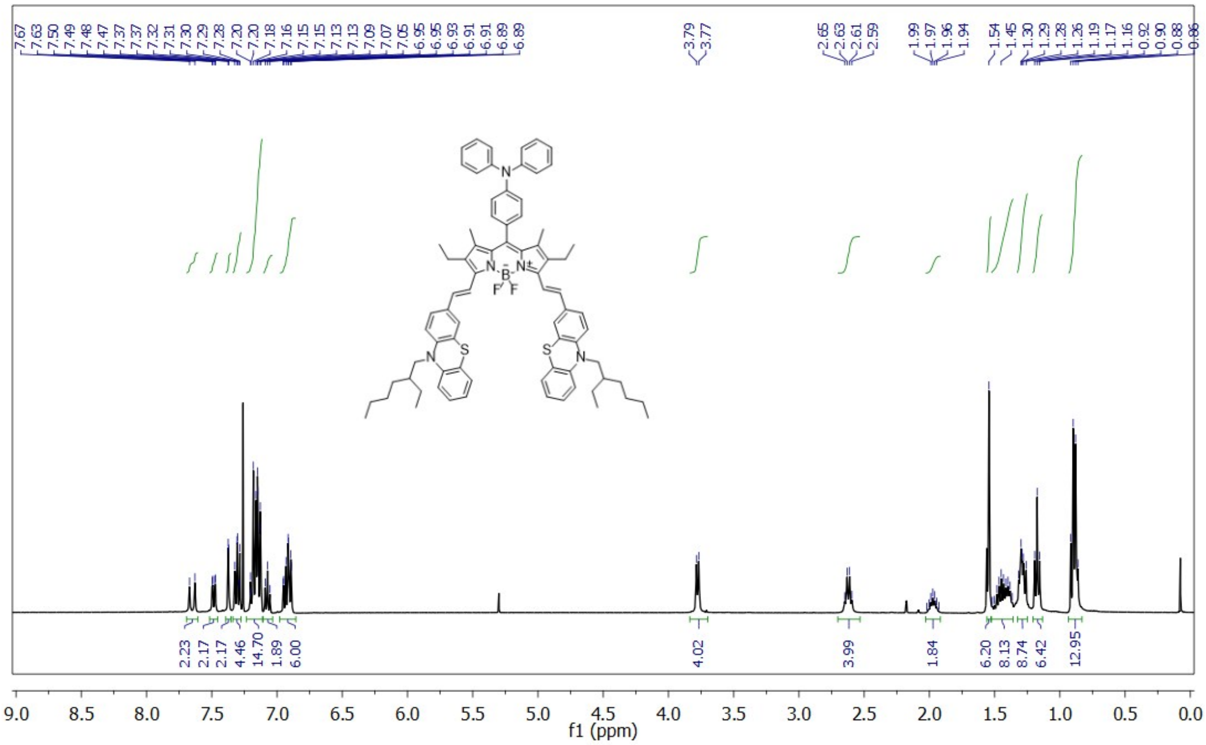

Figure S3: ^{1}H NMR (400 MHz, $CDCl_3$) of **TPA-BDP-PTZ**.

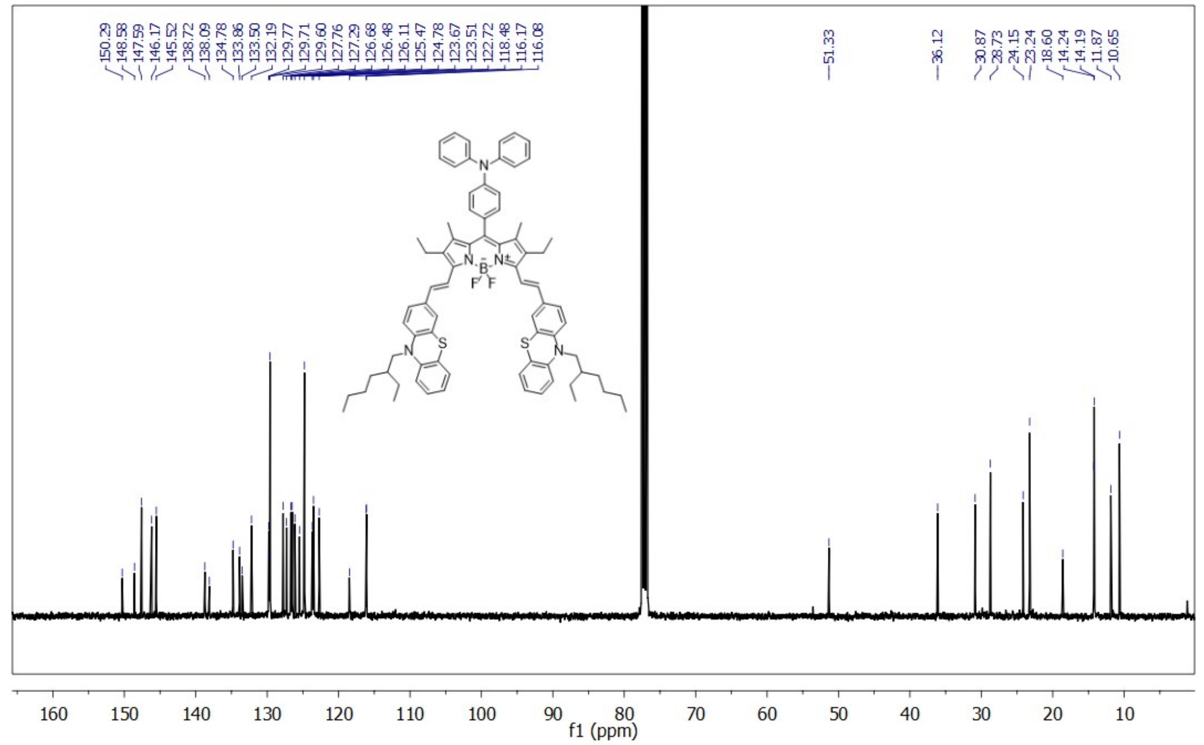

Figure S4: ^{13}C NMR (100 MHz, $CDCl_3$) of **TPA-BDP-PTZ**.

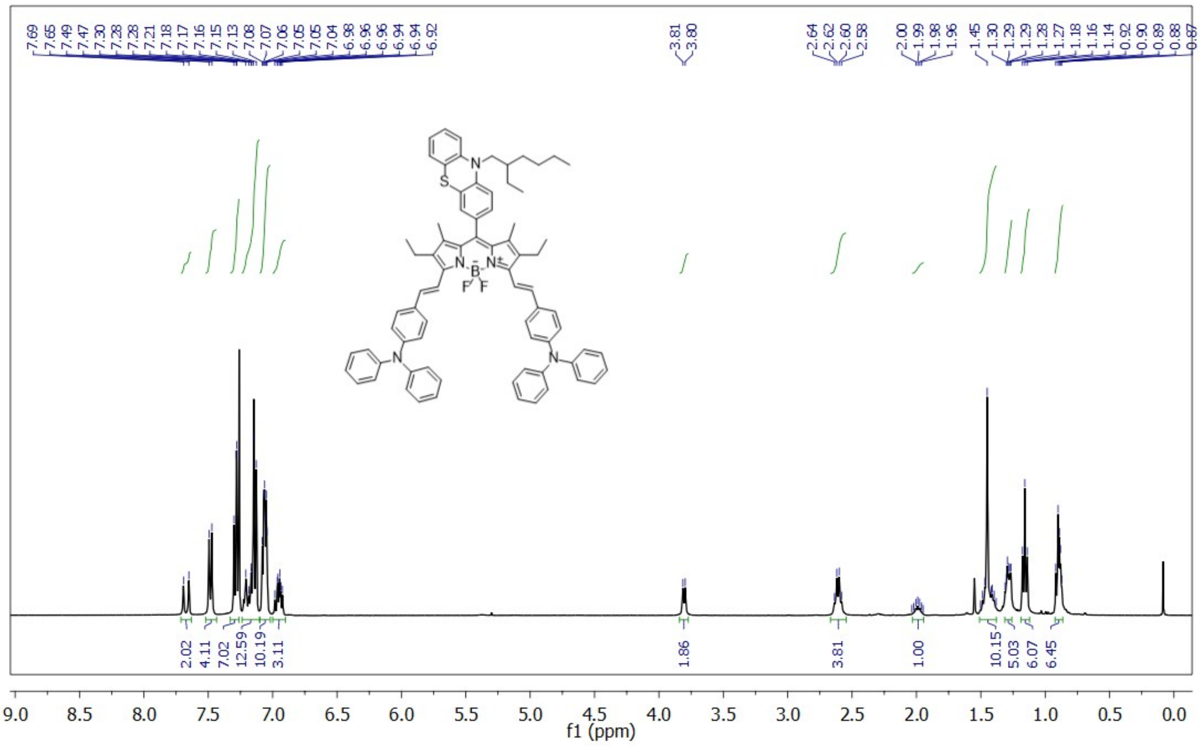

Figure S5: ^{1}H NMR (400 MHz, $CDCl_3$) of **PTZ-TPA-BDP.**

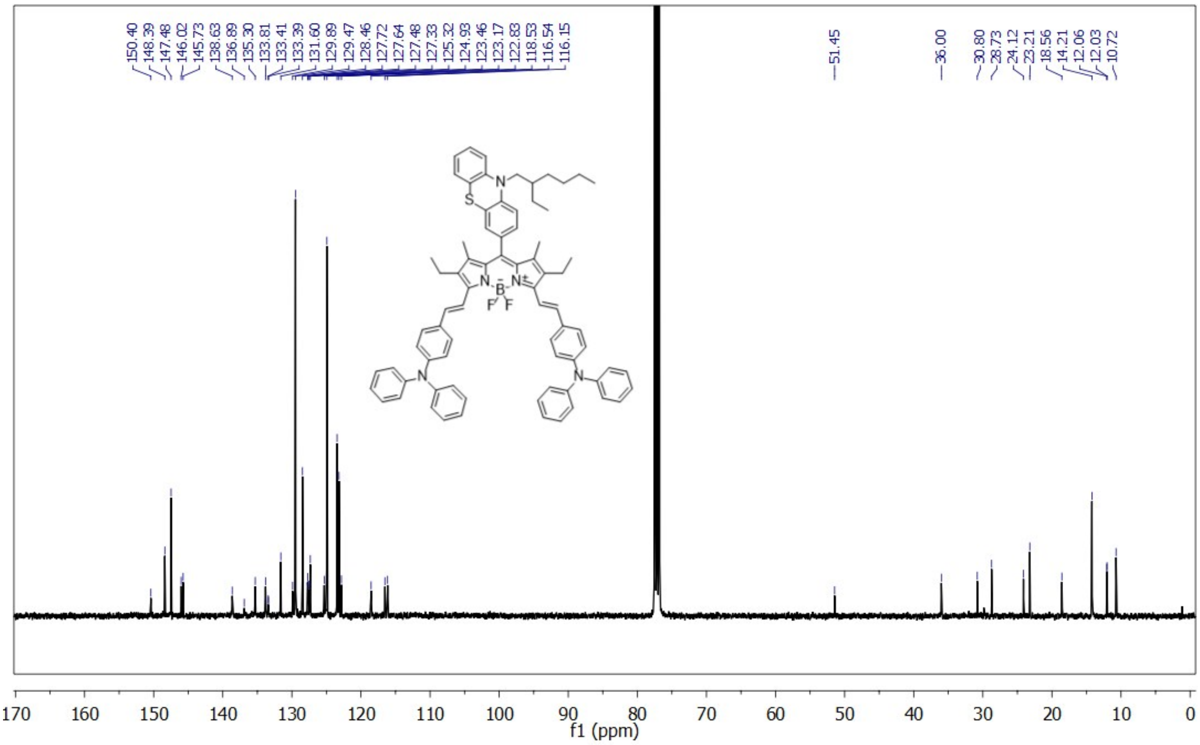

Figure S6: ^{13}C NMR (100 MHz, $CDCl_3$) of **PTZ-TPA-BDP.**

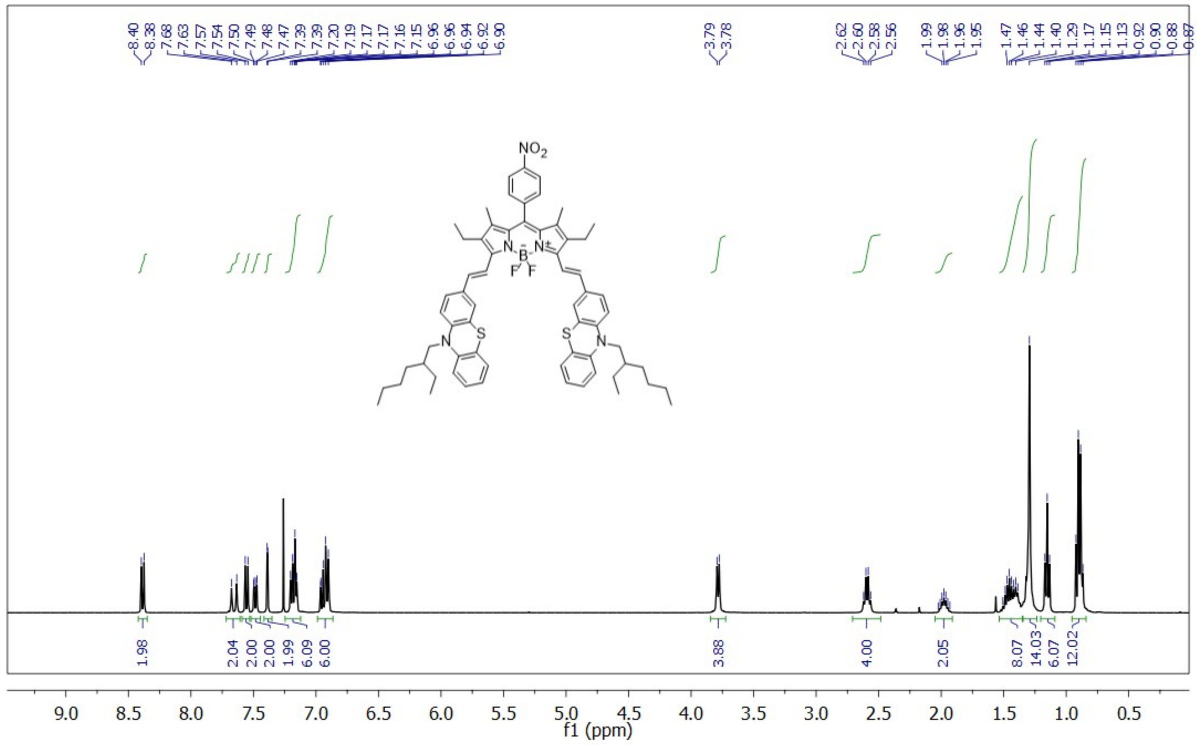

Figure S7: ^{1}H NMR (400 MHz, $CDCl_3$) of **N-PTZ-BDP.**

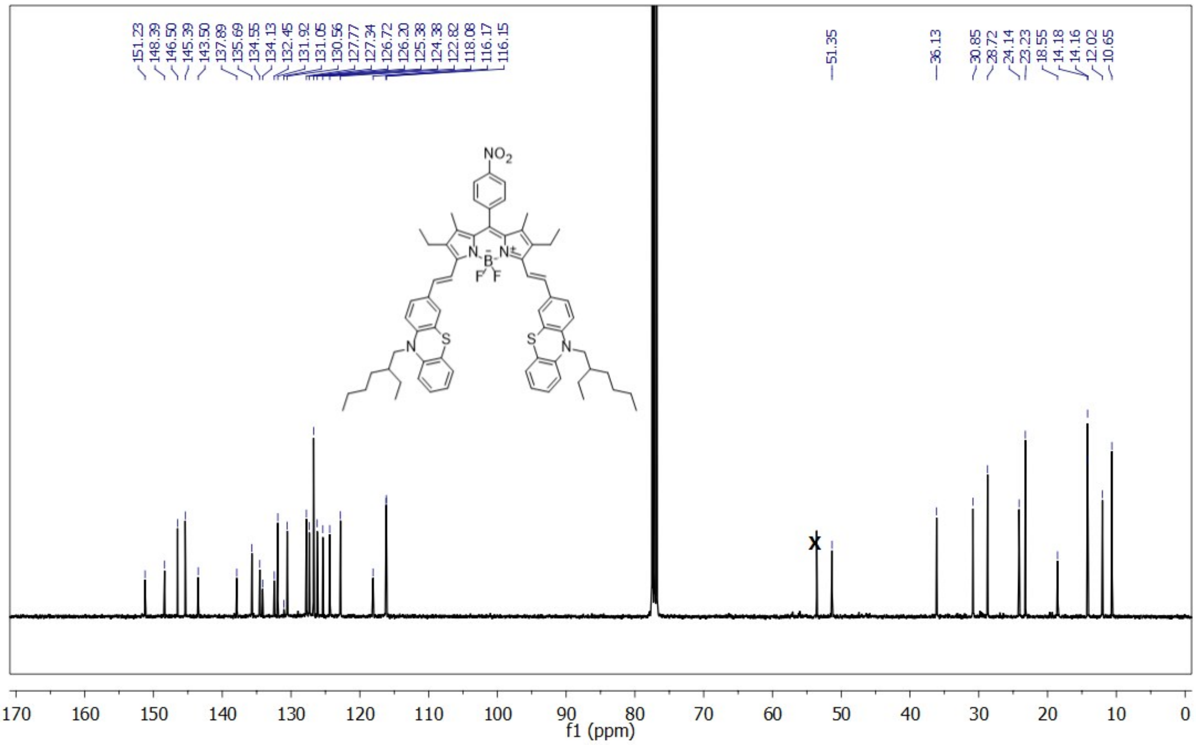

Figure S8: ^{13}C NMR (100 MHz, $CDCl_3$) of **N-PTZ-BDP.**

ESI3 UV-vis absorption spectra.

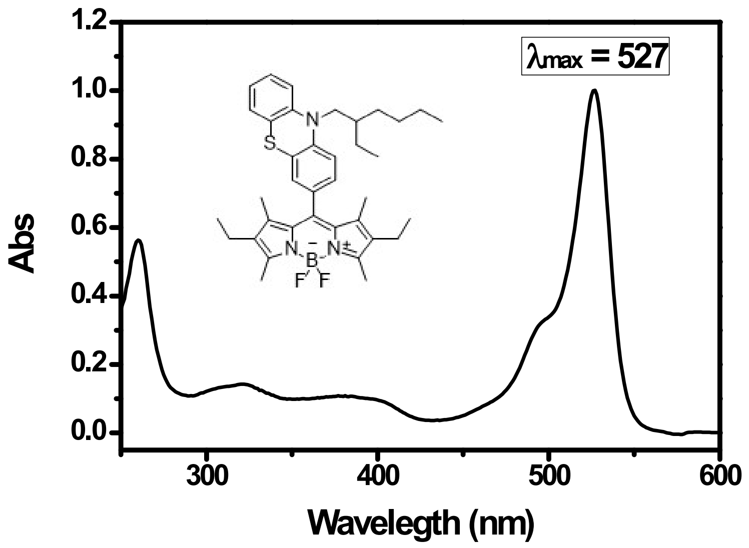

Figure S9: UV-vis absorption spectra of compound **8** recorded in DCM solution (1 x 10^{-5} M).

ESI4 Donor : acceptor weight ratio optimization for different BODIPY donor molecules.

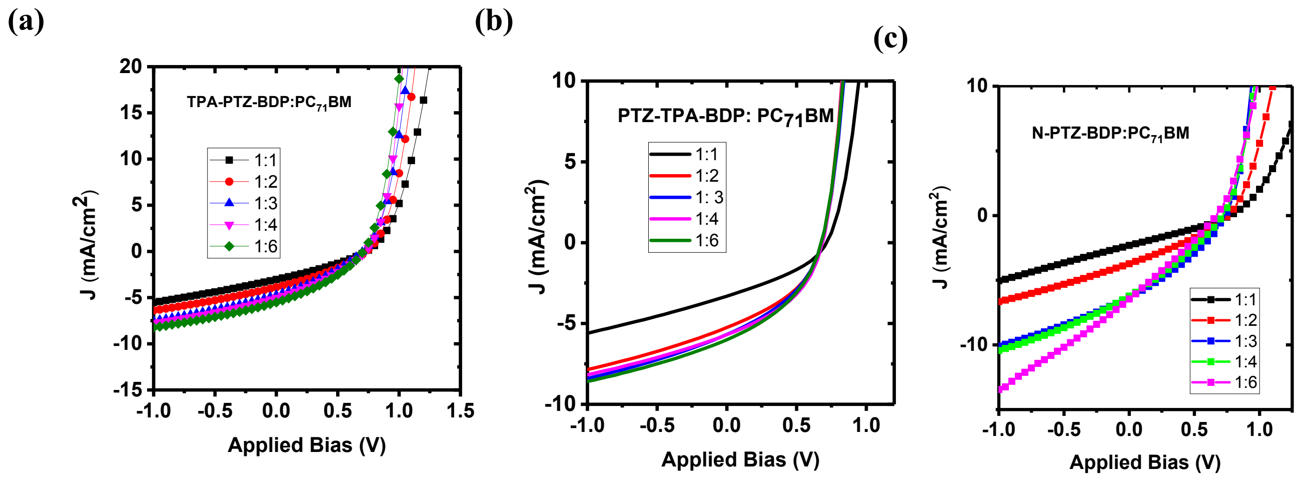

Figure S10: J-V characteristics corresponding to different donor to acceptor ratio for (a) **TPA-PTZ-BDP:$PC_{71}BM$** (b) **PTZ-TPA-BDP:$PC_{71}BM$** and (c) **N-PTZ-BDP:$PC_{71}BM$** blends

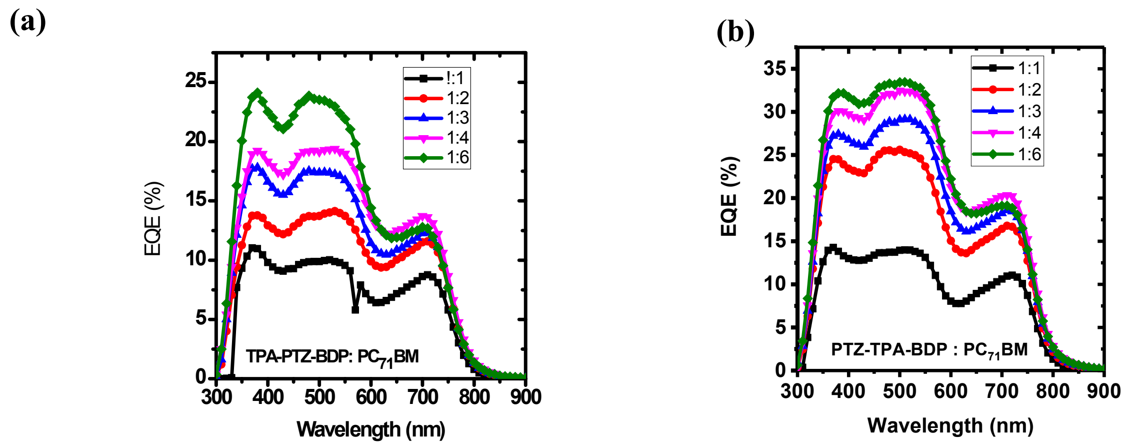

Figure S11: EQE spectra corresponding to different donor to acceptor ratio for (a) **TPA-PTZ-BDP:PC$_{71}$BM** and (b) **PTZ-TPA-BDP:PC$_{71}$BM** blends.

Table S3: Photovoltaic performance parameters (averaged over 8 devices) of the **TPA-PTZ-BDP:PC$_{71}$BM** blend as a function of different donor : acceptor weight ratio

D:A ratio (wt%)	Jsc (mA/cm^2)	Voc (V)	FF (%)	Rsh (Ohmcm^2)	Rs (Ohmcm^2)	PCE avg. (%)	PCE Best (%)
1:1	2.80±0.12	0.711±0.036	30.2±0.5	352±20.9	9.08±2.71	0.60±0.04	0.68
1:2	3.83±0.07	0.712±0.018	31.6±0.7	299±12.9	6.44±1.73	0.86±0.05	0.92
1:3	4.55±0.17	0.669±0.047	31.5±1.1	241±23	4.79±1.31	0.96±0.06	1.07
1:4	4.91±0.14	0.708±0.015	31.7±0.6	249±11	3.61±1.20	1.10±0.06	1.18
1:6	5.09±0.36	0.687±0.015	32.5±1.1	247±18.5	4.35±1.45	1.14±0.11	1.33

Table S4: Photovoltaic performance parameters (averaged over 8 devices) of the **PTZ-TPA-BDP:PC$_{71}$BM** blend as a function of different donor : acceptor weight ratio

D:A ratio (wt%)	Jsc (mA/cm^2)	Voc (V)	FF (%)	Rsh (Ohmcm^2)	Rs (Ohmcm^2)	PCE avg. (%)	PCE Best (%)
1:1	3.20±0.08	0.711±0.018	35.3±0.3	377±17	3.74±0.82	0.80±0.02	0.85
1:2	5.02±0.13	0.681±0.010	40.2±0.2	306±12	2.76±1.17	1.37±0.04	1.44
1:3	5.47±0.17	0.686±0.011	39.5±0.5	291±20	2.33±1.14	1.48±0.02	1.51
1:4	5.70±0.11	0.672±0.008	40.8±0.8	289±13	2.11±0.32	1.56±0.04	1.62
1:6	5.84±0.10	0.674±0.006	38.8±0.5	274±11	2.04±0.22	1.53±0.03	1.60

Table S5: Photovoltaic performance parameters (averaged over 8 devices) of the **N-PTZ-BDP:PC$_{71}$BM** blend as a function of different donor : acceptor weight ratio

Donor to acceptor ratio	J_{sc} (mA/cm^2)	V_{oc} (V)	FF (%)	PCE Avg. (%)	PCE best (%)
1:1	2.17±0.11	0.776±0.008	28.5±0.7	0.48±0.03	0.52
1:2	3.62±0.18	0.715±0.006	31.4±1.8	0.81±0.08	0.87
1:3	5.83±0.44	0.699±0.114	33.4±6.7	1.33±0.14	1.51
1:4	5.88±0.38	0.718±0.007	30.1±1.0	1.27 ±0.11	1.34
1:6	5.99±0.41	0.686±0.026	25.6±4.2	1.06±0.24	1.19

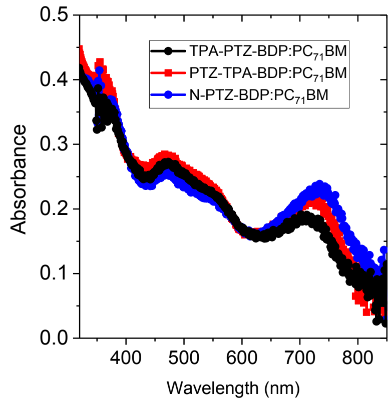

Figure S12: Absorbance spectra comparison (for optimized donor:acceptor ratio) for blends of three different BDP molecule with **PC$_{71}$BM**.

ESI5 DFT.

Coordinates of optimised B3lyp/631g(d,p) geometry of TPA-PTZ-BDP.

C	2.53159417	-1.20443026	-0.22019663
C	2.53159768	1.20444921	0.22016882
C	3.22653360	0.00000790	-0.00001075
C	2.99549505	-2.53883566	-0.48460686
C	1.86700260	-3.34508187	-0.61502878
C	0.72204104	-2.50597093	-0.42753568
C	0.72204814	2.50599684	0.42749943
C	2.99550245	2.53885207	0.48458334
C	1.86701346	3.34510545	0.61498790
B	0.21198152	0.00001514	-0.00002429
F	-0.58783009	0.17555152	-1.12938542
F	-0.58783672	-0.17551763	1.12933259
N	1.14349593	1.23453165	0.19106173
N	1.14349178	-1.23450534	-0.19110514
C	-0.68708013	2.82395885	0.37474250
H	-1.28202770	2.10293122	-0.17533482
C	-0.68708580	-2.82393633	-0.37477220
H	-1.28203789	-2.10290120	0.17529055
C	-1.29552257	-3.89145752	-0.94589940
H	-0.71086476	-4.57140718	-1.55791328
C	-1.29552734	3.89145935	0.94589800
H	-0.71087927	4.57139431	1.55793671
C	4.41307459	-3.02244482	-0.58983896
H	4.95222555	-2.53441806	-1.40743399
H	4.98413245	-2.82561891	0.32221543
H	4.43687647	-4.09865142	-0.77465190
C	4.41308325	3.02245546	0.58982432
H	4.95222822	2.53442530	1.40742112
H	4.98414503	2.82562910	-0.32222763
H	4.43688791	4.09866174	0.77463902
C	1.86221912	-4.83229757	-0.84786168

H	0.97056931	-5.26819854	-0.38612487
H	2.71433941	-5.28220906	-0.32525851
C	1.86224151	4.83232376	0.84780444
H	0.97060985	5.26823110	0.38604008
H	2.71438020	5.28221972	0.32521763
C	1.92795362	-5.23605636	-2.33295907
H	2.84761927	-4.86693360	-2.79784877
H	1.90584610	-6.32550231	-2.44438089
H	1.08996994	-4.81872107	-2.90054730
C	1.92794207	5.23609674	2.33289932
H	2.84758964	4.86696526	2.79781782
H	1.90584756	6.32554416	2.44430914
H	1.08993641	4.81877959	2.90046867
C	-2.71262946	4.22755286	0.86182671
C	-3.63543855	3.52343661	0.06019900
C	-3.20415681	5.31669912	1.59995079
C	-4.97849828	3.87048930	0.03280353
H	-3.30618386	2.68315304	-0.54177441
C	-4.54285495	5.69282190	1.54998241
H	-2.51919416	5.89128539	2.21767540
C	-5.46313718	4.97053567	0.77546859
H	-4.86711595	6.55989491	2.11304026
C	-2.71262170	-4.22756233	-0.86182112
C	-3.63543903	-3.52343255	-0.06021483
C	-3.20413699	-5.31673447	-1.59991491
C	-4.97849556	-3.87049724	-0.03281231
H	-3.30619343	-2.68312936	0.54173613
C	-4.54283170	-5.69286852	-1.54993883
H	-2.51916749	-5.89133195	-2.21762148
C	-5.46312252	-4.97056884	-0.77544783
H	-4.86708313	-6.55996095	-2.11297229
C	-7.27393007	-4.13208595	1.36821860
C	-7.95583910	-4.02099017	2.57940821
C	-7.53384141	-5.20779759	0.49595972

C	-8.93474536	-4.95254650	2.92953518
H	-7.72521640	-3.19022933	3.23933204
C	-8.49639059	-6.15555301	0.87497555
C	-9.20107730	-6.01685327	2.07212815
H	-9.47496918	-4.84683466	3.86485764
H	-8.69675450	-7.00974500	0.23920025
H	-9.95001784	-6.75788536	2.33488156
S	-6.13418605	-2.85693006	0.87059794
S	-6.13417753	2.85693562	-0.87063623
N	-6.83069031	-5.30745418	-0.72827930
C	-7.35702096	-6.19494364	-1.75361036
H	-7.12768226	-7.25729073	-1.57949562
H	-8.44146172	-6.07887544	-1.80044880
H	-6.94179610	-5.90567006	-2.72091806
N	-6.83070788	5.30740947	0.72830679
C	-7.27393330	4.13209398	-1.36822359
C	-7.95583947	4.02102431	-2.57941718
C	-7.53385628	5.20777946	-0.49593597
C	-8.93475409	4.95258069	-2.92952056
H	-7.72520783	3.19028350	-3.23936312
C	-8.49641366	6.15553602	-0.87492794
C	-9.20109736	6.01686190	-2.07208530
H	-9.47497553	4.84688893	-3.86484670
H	-8.69678652	7.00970923	-0.23913020
H	-9.95004441	6.75789404	-2.33481994
C	-7.35704863	6.19486495	1.75366199
H	-7.12772110	7.25721924	1.57957675
H	-8.44148813	6.07878389	1.80049680
H	-6.94182108	5.90556904	2.72096185
C	4.72108519	0.00000477	-0.00000479
C	5.44155442	-0.21996961	1.18155823
C	5.44156479	0.21997617	-1.18156201
C	6.83366053	-0.21123471	1.18880564
H	4.90402984	-0.38497572	2.11091747

C	6.83367095	0.21123549	-1.18879831
H	4.90404834	0.38498465	-2.11092551
C	7.55327290	-0.00000111	0.00000653
H	7.37072289	-0.36898803	2.11767870
H	7.37074141	0.36898677	-2.11766703
N	8.96941255	-0.00000391	0.00001237
C	9.68729287	-0.90711820	0.83031568
C	9.29311774	-2.25083177	0.92789193
C	10.80552351	-0.47001815	1.55730018
C	9.99795453	-3.13170173	1.74574190
H	8.43546038	-2.59716821	0.36092905
C	11.51525355	-1.36285263	2.35772983
H	11.11259142	0.56815615	1.48833961
C	11.11459266	-2.69623536	2.46190150
H	9.67954027	-4.16818845	1.81041030
H	12.37855003	-1.00849553	2.91361020
H	11.66557344	-3.38710359	3.09238785
C	9.68730340	0.90710747	-0.83028498
C	9.29313430	2.25082257	-0.92786464
C	10.80553859	0.47000310	-1.55725991
C	9.99798162	3.13168980	-1.74570852
H	8.43547350	2.59716236	-0.36090911
C	11.51527906	1.36283487	-2.35768338
H	11.11260195	-0.56817239	-1.48829679
C	11.11462420	2.69621915	-2.46185845
H	9.67957191	4.16817776	-1.81037962
H	12.37857898	1.00847443	-2.91355628
H	11.66561313	3.38708527	-3.09234001

Coordinates of optimised B3lyp/631g(d,p) geometry of PTZ-TPA-BDP.

C	-3.57194290	-1.46088585	-0.56393762
C	-3.71863427	0.98157811	-0.42250481
C	-4.33665738	-0.27920170	-0.52330448

C	-3.95601498	-2.84064659	-0.68303482
C	-2.78364631	-3.59248624	-0.67269798
C	-1.69087687	-2.67628600	-0.54313117
C	-1.99661768	2.40709309	-0.25691427
C	-4.26889093	2.31056059	-0.38576911
C	-3.19801011	3.19282511	-0.27649037
B	-1.35821353	-0.11960675	-0.20409519
F	-0.83110132	-0.16273005	1.07884788
F	-0.32488299	-0.00051343	-1.14742974
N	-2.33696758	1.08898584	-0.34665464
N	-2.18638536	-1.40721399	-0.49387614
C	-0.60562877	2.77866745	-0.17065027
H	0.07606416	1.94468332	-0.27716470
C	-0.26485840	-2.89959695	-0.52375178
H	0.32038084	-2.01949453	-0.76251912
C	0.37703330	-4.05634138	-0.22355605
H	-0.19945289	-4.92511858	0.07802664
C	-0.07766155	4.01549498	0.01703882
H	-0.73175588	4.87393146	0.12002377
C	-5.33921381	-3.40978532	-0.81451758
H	-5.95623787	-3.19088047	0.06240812
H	-5.87229554	-3.00588497	-1.67973994
H	-5.29718899	-4.49552439	-0.92386982
C	-5.71585561	2.70465722	-0.46247618
H	-6.18112984	2.36775190	-1.39321720
H	-6.30307196	2.27483380	0.35405651
H	-5.82434462	3.78979036	-0.41480005
C	-2.69048088	-5.08744540	-0.82387478
H	-1.75022169	-5.34369404	-1.32267335
H	-3.48364617	-5.43161567	-1.49749824
C	-3.29358476	4.69514300	-0.22802695
H	-2.63370036	5.08681663	0.55367102
H	-4.30232466	4.98000246	0.08644484
C	-2.80258169	-5.86154642	0.50325293

H	-3.76786713	-5.67485491	0.98427443
H	-2.71032171	-6.94024588	0.33680397
H	-2.02537628	-5.55856196	1.21205783
C	-2.98224040	5.38193874	-1.57104764
H	-3.69179197	5.06203863	-2.34047939
H	-3.04992991	6.47128270	-1.47881987
H	-1.97804746	5.13185908	-1.92489500
C	1.81986504	-4.26339729	-0.22556501
C	2.33370601	-5.51748156	0.15975914
C	2.75610036	-3.27402274	-0.59499759
C	3.69638763	-5.78221206	0.17172438
H	1.64137456	-6.30640366	0.44312544
C	4.11884454	-3.52368486	-0.57447647
H	2.41348981	-2.28709543	-0.88854331
C	4.61672660	-4.78570060	-0.19458451
H	4.05651914	-6.76321010	0.46151628
H	4.81457248	-2.73883178	-0.85056551
C	1.34114696	4.33296563	0.10940200
C	1.73004082	5.66697678	0.34483137
C	2.37402359	3.37920938	-0.02030738
C	3.06286405	6.03647892	0.46072732
H	0.96157012	6.42771998	0.45787777
C	3.70797315	3.73984875	0.07928127
H	2.13121345	2.34021100	-0.21773260
C	4.08017056	5.07586702	0.32880871
H	3.32389305	7.07016037	0.65899878
H	4.47934591	2.98666043	-0.03817368
C	-5.82737379	-0.36946796	-0.58833718
C	-6.58484287	-0.47995023	0.58544335
C	-6.50239478	-0.34419994	-1.81153595
C	-7.97267323	-0.59773698	0.53629096
H	-6.08528690	-0.49158472	1.54942762
C	-7.89451645	-0.42332493	-1.86649230
H	-5.93777160	-0.24756360	-2.73393610

C	-8.65588461	-0.56731284	-0.69656511
H	-8.38482732	-0.36949231	-2.83123547
C	-10.38482321	-0.01742110	1.60080802
C	-11.12984032	0.61988721	2.59191272
C	-10.83552188	-0.04232984	0.26640159
C	-12.35746311	1.20903748	2.28472405
H	-10.74969378	0.63727973	3.60877330
C	-12.05500720	0.58258162	-0.03469089
C	-12.81490266	1.18479354	0.96981337
H	-12.94204295	1.68464715	3.06554920
H	-12.41628609	0.60528599	-1.05598941
H	-13.76185900	1.64867779	0.71096050
S	-8.89125852	-0.88902006	2.03444465
N	-10.06165254	-0.69132307	-0.72570046
C	-10.69780705	-1.01065110	-1.99437430
H	-10.78618933	-0.15009040	-2.67486707
H	-10.12005683	-1.78973227	-2.49566304
H	-11.69825643	-1.40184186	-1.80041166
N	5.44280322	5.43757262	0.44255935
N	6.00872792	-5.04027407	-0.18190033
C	6.36735250	4.56419829	1.08227641
C	7.64461879	4.36139291	0.53631225
C	6.01898713	3.89970671	2.26883960
C	8.55317861	3.51722177	1.17117811
H	7.91725578	4.86762020	-0.38364429
C	6.92818847	3.04303569	2.88593694
H	5.03624487	4.05795669	2.69991548
C	8.20096956	2.84929619	2.34560379
H	9.53731970	3.37075914	0.73531796
H	6.64224040	2.53555219	3.80271032
H	8.90873169	2.18607176	2.83313658
C	5.89569962	6.69248419	-0.05383712
C	5.48273409	7.15015816	-1.31521094
C	6.76593389	7.48667711	0.70910763

C	5.92513770	8.38153420	-1.79381556
H	4.81675292	6.53635399	-1.91238244
C	7.21692953	8.70863104	0.21407774
H	7.08377298	7.13983809	1.68657976
C	6.79691668	9.16643619	-1.03622502
H	5.59696462	8.72122286	-2.77201159
H	7.88990028	9.31142822	0.81713707
H	7.14490772	10.12210200	-1.41591937
C	6.84766346	-4.49455961	-1.19369874
C	8.09754493	-3.94885977	-0.86091121
C	6.44187071	-4.49998495	-2.53774149
C	8.92351727	-3.42888171	-1.85506925
H	8.41345301	-3.93686781	0.17676282
C	7.26741082	-3.96103480	-3.52223349
H	5.48040508	-4.92673357	-2.80262125
C	8.51373194	-3.42658275	-3.18987971
H	9.88749677	-3.01018466	-1.58049777
H	6.93812694	-3.97290574	-4.55728358
H	9.15697969	-3.01367308	-3.96073997
C	6.57786588	-5.86088182	0.83255482
C	6.19757529	-5.70182559	2.17461784
C	7.53263763	-6.83624794	0.50493680
C	6.75612040	-6.51020522	3.16243314
H	5.46607993	-4.94415907	2.43482832
C	8.09864308	-7.62756087	1.50243791
H	7.82621755	-6.96633596	-0.53124015
C	7.71216842	-7.47398723	2.83523986
H	6.45148914	-6.37394610	4.19613994
H	8.83626251	-8.37775392	1.23205676
H	8.15035010	-8.09683583	3.60893106

Coordinates of optimised B3lyp/631g(d, p) geometry of N-PTZ-BDP.

C	4.11012327	1.22151764	0.11183761
C	4.11011516	-1.22154824	-0.11182964
C	4.79978515	-0.00001715	0.00000429
C	4.57379126	2.57621352	0.24828106
C	3.44506676	3.38887660	0.31316002
C	2.29949031	2.53408165	0.21197437
C	2.29947083	-2.53409814	-0.21196461
C	4.57377127	-2.57624865	-0.24827986
C	3.44503967	-3.38890228	-0.31315313
B	1.79102173	-0.00000610	0.00000275
F	0.99210206	-0.08206049	1.13976794
F	0.99210436	0.08205490	-1.13976371
N	2.72239786	-1.24690126	-0.08829338
N	2.72240682	1.24688170	0.08830066
C	6.29487776	-0.00001755	0.00000132
C	7.00510851	0.06493328	-1.20841947
C	7.00511332	-0.06496096	1.20841967
C	8.39731469	0.06569145	-1.21687088
H	6.45941976	0.11693191	-2.14506501
C	8.39731961	-0.06571244	1.21686592
H	6.45942839	-0.11695992	2.14506746
C	9.07234192	-0.00000942	-0.00000381
H	8.96335643	0.11624516	-2.13845257
H	8.96336495	-0.11626197	2.13844568
C	0.88904923	-2.83947779	-0.15259167
H	0.28872076	-2.06252980	0.30749261
C	0.88907103	2.83947176	0.15259845
H	0.28873770	2.06252608	-0.30748339
C	0.28278346	3.95658820	0.62390569
H	0.87578857	4.70611627	1.13882122
C	0.28275462	-3.95658790	-0.62390525
H	0.87575540	-4.70611695	-1.13882486

C	5.99088485	3.07061373	0.29942649
H	6.51620969	2.71927952	1.19343886
H	6.57766548	2.74046114	-0.56216157
H	6.01442901	4.16218283	0.31692083
C	5.99085903	-3.07066460	-0.29943405
H	6.51618281	-2.71933580	-1.19344909
H	6.57764833	-2.74052038	0.56215103
H	6.01439011	-4.16223406	-0.31692975
C	3.43841987	4.89098643	0.41078949
H	2.54370637	5.28173486	-0.08402825
H	4.28656096	5.29316539	-0.15524529
C	3.43837971	-4.89101206	-0.41078263
H	2.54366751	-5.28175310	0.08404323
H	4.28652273	-5.29319804	0.15524450
C	3.51167160	5.42754784	1.85297709
H	4.43365757	5.10180895	2.34466290
H	3.49074058	6.52245160	1.86455819
H	2.67630823	5.06450724	2.46024276
C	3.51161164	-5.42757371	-1.85297099
H	4.43359388	-5.10184048	-2.34466761
H	3.49067355	-6.52247732	-1.86455189
H	2.67624302	-5.06452775	-2.46022647
C	-1.14062379	-4.26352544	-0.55214667
C	-2.08592697	-3.43728010	0.09172516
C	-1.61837036	-5.44303886	-1.14667250
C	-3.43518670	-3.75856772	0.10314343
H	-1.76974476	-2.51850879	0.57401161
C	-2.96460470	-5.79160003	-1.10623464
H	-0.91695616	-6.11042038	-1.64010092
C	-3.90618995	-4.95117775	-0.49246827
H	-3.27874146	-6.72829806	-1.55084240
C	-1.14059290	4.26353440	0.55214472
C	-2.08590185	3.43728879	-0.09171816
C	-1.61833139	5.44305670	1.14665933

C	-3.43515951	3.75858507	-0.10313865
H	-1.76972607	2.51851027	-0.57399498
C	-2.96456346	5.79162633	1.10621894
H	-0.91691253	6.11043848	1.64008072
C	-3.90615468	4.95120402	0.49246163
H	-3.27869368	6.72833076	1.55081764
C	-5.81031328	3.76279157	-1.39163084
C	-6.55715700	3.45195778	-2.52738918
C	-6.04831138	4.95623273	-0.68211837
C	-7.57854873	4.30035741	-2.95766251
H	-6.34293753	2.53226748	-3.06320636
C	-7.05526477	5.81674648	-1.14459682
C	-7.82326273	5.48082440	-2.26098033
H	-8.16832223	4.04056039	-3.83084952
H	-7.24250525	6.75658327	-0.63877170
H	-8.60491711	6.15906627	-2.58958459
S	-4.61089840	2.59960015	-0.77443872
S	-4.61091753	-2.59958228	0.77445662
N	-5.27974494	5.25863856	0.46793223
C	-5.77446670	6.28152535	1.37652538
H	-5.58181353	7.30881164	1.03204606
H	-6.85155720	6.15460277	1.50049372
H	-5.30202625	6.14913949	2.35169175
N	-5.27978214	-5.25860376	-0.46794083
C	-5.81033915	-3.76277248	1.39163811
C	-6.55717960	-3.45194561	2.52740048
C	-6.04834551	-4.95620484	0.68211365
C	-7.57857620	-4.30034325	2.95766625
H	-6.34295367	-2.53226218	3.06322689
C	-7.05530372	-5.81671701	1.14458445
C	-7.82329835	-5.48080159	2.26097226
H	-8.16834699	-4.04055156	3.83085665
H	-7.24255050	-6.75654745	0.63874994
H	-8.60495654	-6.15904193	2.58957050

C	-5.77451073	-6.28147883	-1.37654351
H	-5.58186449	-7.30876962	-1.03207374
H	-6.85160033	-6.15454784	-1.50051082
H	-5.30206917	-6.14908710	-2.35170855
O	11.11542966	-0.06024102	1.08849151
O	11.11542532	0.06023878	-1.08850648
N	10.54462092	-0.00000535	-0.00000680
